# Traces of Introgression from cAus into Tropical Japonica Observed in African Upland Rice Varieties

**DOI:** 10.1186/s12284-023-00625-4

**Published:** 2023-02-28

**Authors:** Abdoulaye Beye, Claire Billot, Joëlle Ronfort, Kenneth L. McNally, Diaga Diouf, Jean Christophe Glaszmann

**Affiliations:** 1 CIRAD, UMR AGAP Institut, 34398 Montpellier, France; 2 UMR AGAP Institut, CIRAD, INRAE, Institut Agro, Université de Montpellier, 34398 Montpellier, France; 3 International Rice Research Institute, DAPO Box 7777, Metro Manila, 1301 The Philippines; 4 Laboratoire Campus de Biotechnologies Végétales, Département de Biologie Végétale, Faculté Des Sciences Et Techniques, Université Cheikh Anta Diop, 10700 Dakar-Fann, Dakar, Senegal

**Keywords:** Rice, *Oryza sativa*, Africa, Upland, Introgression, Japonica, cAus

## Abstract

**Background:**

Asian rice *Oryza sativa*, first domesticated in East Asia, has considerable success in African fields. When and where this introduction occurred is unclear. Rice varieties of Asian origin may have evolved locally during and after migration to Africa, resulting in unique adaptations, particularly in relation to upland cultivation as frequently practiced in Africa.

**Methods:**

We investigated the genetic differentiation between Asian and African varieties using the 3000 Rice Genomes SNP dataset. African upland cultivars were first characterized using principal component analysis among 292 tropical Japonica accessions from Africa and Asia. The particularities of African accessions were then explored using two inference techniques, PCA-KDE for supervised classification and chromosome painting, and ELAI for individual allelic dosage monitoring.

**Key Results:**

Ambiguities of local differentiation between Japonica and other groups pointed at genomic segments that potentially resulted from genetic exchange. Those specific to West African upland accessions were concentrated on chromosome 6 and featured several cAus introgression signals, including a large one between 17.9 and 21.7 Mb. We found iHS statistics in support of positive selection in this region and we provide a list of candidate genes enriched in GO terms that have regulatory functions involved in stress responses that could have facilitated adaptation to harsh upland growing conditions.

**Supplementary Information:**

The online version contains supplementary material available at 10.1186/s12284-023-00625-4.

## Background

Rice (*Oryza sativa* L.) domestication has started over 10,000 years ago inYangtze River Valley. With an annual expansion rate of 0.39 percent during the last 30 years (Van Nguyen and Ferrero 2006), it currently constitutes a staple diet for over half of the world population and it covers an area of more than 150 million hectares. The wide distribution of this species outside its origin reflects its importance for human and the ability of the crop to adapt to different environments.

One particular path of interest is its migration from Asia to Africa, which should involve adaptation to severely dry conditions for upland rice cultivation (Bernier et al. 2008). Both continents have their own species of cultivated rice: *Oryza sativa* (Asian rice) and *Oryza glaberrima* (African rice). Despite this double origin, it is notable that most rice grown in Africa is *O. sativa* while no *O. glaberrima* rice has been found in Asia. This contrasts with patterns observed for sorghum and pearl millet, for example, which are both of African origin and have disseminated throughout Asia for millennia (Fuller and Boivin 2009). Migration of Asian rice to Africa is thought to have followed several routes at several times (Gilbert 2015).

Human migration has brought Asian rice to Africa via Madagascar. The evidence of Indonesian colonisation in Madagascar is unequivocal. Previous investigations carried out in genetics, linguistics, archaeology, and ethnography support the connections between the Indonesian and Malagasy peoples (Cox et al. 2012; Serva et al. 2012; Vérin and Wright 1999). The introduction of the Asian rice by the East coast of Africa occurred during the colonization of the island of Madagascar (Beaujard 2011; Gilbert 2015). The most recent introductions of Asian rice into Africa before modern era are credited to the European maritime adventurers and traders, who would have brought Asian rice to the West African coasts on their way back from Asia between the end of the 15th and the beginning of the sixteenth century (Bezançon 1995). The traces that rice introduction may have left in the genetic composition of African materials have not been thoroughly studied since De Kochko's early work with isozymes (De Kochko 1987), and the most detailed investigations have been concentrated on Madagascar (Ahmadi et al. 2021; Mather et al. 2010).

The early work of Kato in 1928 based on morphological and serological characteristics (Richharia et al. 1962) confirmed the empirical classification of rice varieties in China and identified two subspecies, Japonica (Geng) and Indica (Hsien) (Oka 1988). The distinction also involves F1 hybrid sterility and differentiated patterns of adaptation. Molecular markers further refined the classification of rice varieties, starting with isozymes (Glaszmann 1987). Current work involves novel genome sequencing technologies and recently delivered high quality sequencing of more than three thousand genomes of rice, providing access to millions of SNP markers (The 3000 rice genomes project, 2014). Nine distinct clusters/genetic groups have been used to classify the three thousand rice genomes (3 K-RGs). Most of these subgroups depict global geographic patterns. *O. sativa* Indica subspecies is subdivided into four subgroups: XI-1A (East Asia), XI-1B (modern cultivars), XI-2 (South Asia), and XI-3 (South East Asia). Japonica is classified as tropical (GJ-trp), subtropical (GJ-strp) and temperate (GJ-tmp). Two additional clusters are the circum-Aus (cA) ecotype and circum-Basmati (cB) group (Wang et al. 2018a, b), localized along the Himalayan foothills. Recent studies using data from 3000 genomes in addition to 178 newly resequenced landrace accessions refined the description of diversity within Indica and Japonica and revealed steps and routes for the evolution and dissemination of both cultivar groups during their dissemination from the centres of domestication and diversification within Asia (Gutaker et al. 2020).

While this classification appears valid for *O. sativa* varieties worldwide, there are numerous regions where several types coexist and where hybridization is likely to occur among them.

By accessing to the results of the 3 K-RG rice genome resequencing project, we could work on a massive dataset on sequence diversity. As can be seen in the paper presenting this dataset (Wang et al. 2018a, b), there are numerous approaches to query such a large amount of data. In this work, we addressed the expansion of Asian rice to upland cultivation areas in Africa. We observed a reduction of diversity considering the Japonica component of the genome, but we also found evidence of specific introgression, coming from cAus, which produced novel combinations that have not been observed in Asia. We attempted to assess the contribution to local adaptation of a particular introgression of cA on the q arm of chromosome 6 using genomic selection signature analysis. We pinpointed a non-exhaustive list of candidate genes enriched in functions related to the response to abiotic stress (drought) in the introgressed region of cA.

## Results

### Population Structure Analysis

**Fig. 1:**
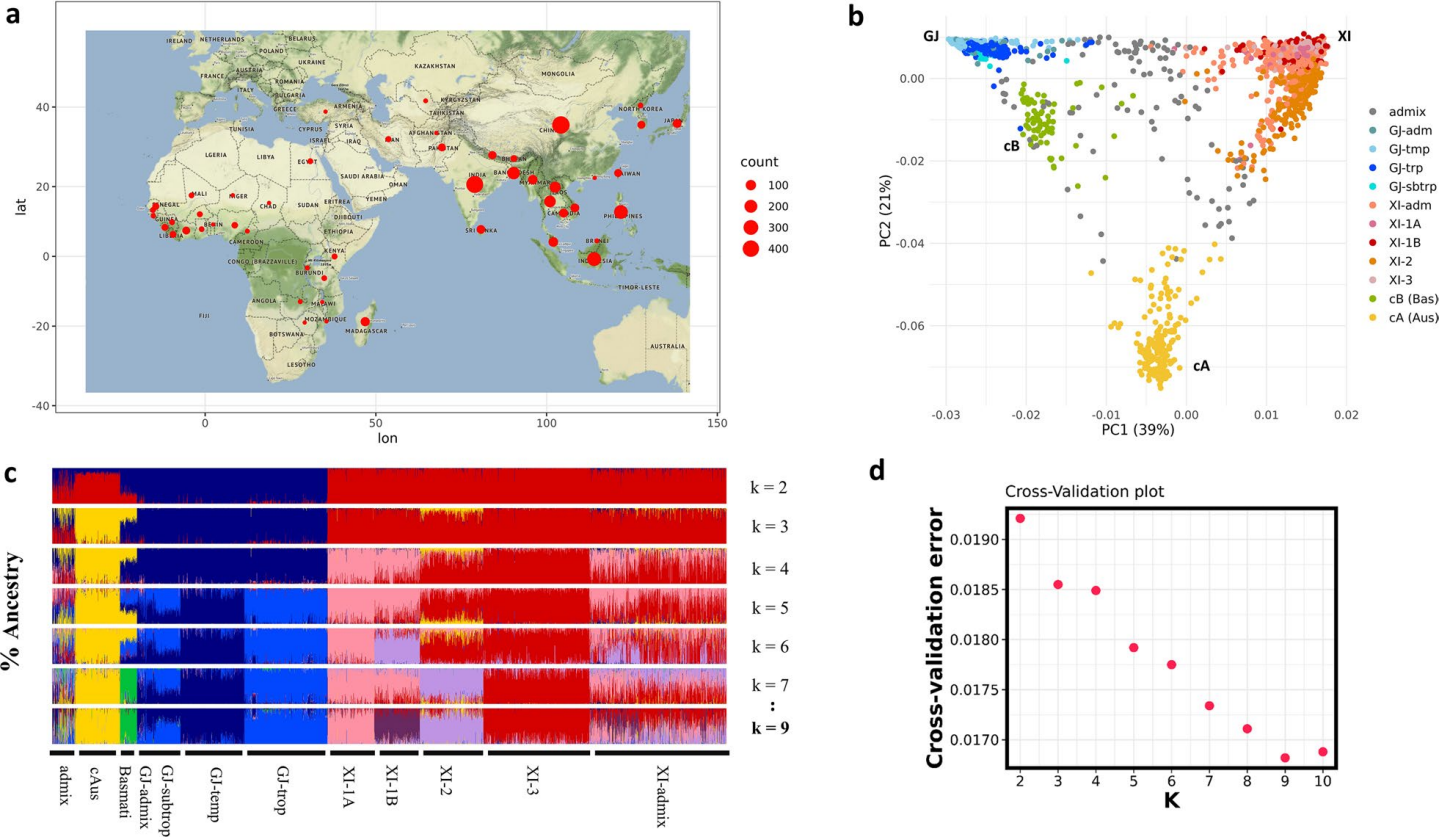
Origin and population structure of African and Asian rice Oryza sativa: **a** Geographic origin and sampled number of accessions is shown from both continents. **b** PCA of 2710 rice accessions from Africa and Asia, along axes 1 and 2. The first axis (39% of the variance) differentiates japonica (GJ-trp, GJ-sbtrp, GJ-tmp, and GJ-adm) from indica (XI-1A, XI-1B, XI-2, XI-3, and XI-adm). The cAus ecotype differs from other groups on the second axis, which explains 21% of the total variance. Basmati accessions (cBas), very close to Japonica, appear to belong to the GJ group but received a contribution from cAus ecotype. **c** Unsupervised hierarchical clustering based on 3023 accessions. Each bar represents an accession. The colours indicate a genetic cluster obtained for K values ranging from 2 to 10. *K* values from 2 to 9 (but not *k* = 8, because no significant cluster were observed) are shown. *K* = 9 has the lowest cross-validation error value. Clusters were assigned to varietal groups as described in Wang et al. 2018a, b. The colours blue, red and yellow correspond to the 3 reference clusters japonica, indica and circum-aus respectively. **d** Cross-validation (CV) error in ADMIXTURE model-based clustering. From *K* = 2 to *K* = 10, *K* = 9 have the lowest error

We first performed global comparisons between accessions from Asia and those from Africa using a Principal Component Analysis (PCA) (Fig. 1b). A total of 2710 individuals were studied, with a total of 7.315.477 unrelated SNPs spread across the entire genome. The accessions are well separated in three main clusters reflecting well-known genetic groups on the first two axes. On the first axis (39% of variance), accessions representing the subspecies Japonica (GJ), represented by the blue colours are differentiated from the subspecies Indica (XI) accessions, represented by varied shades of red–orange. On the second axis (21% of the variance), a third group, composed of the cA ecotype, appears separated from the GJ and XI subspecies. On the second component, the basmati rice varieties seem to be more related to the GJ group. There is no dimensional variation among the three GJ subgroups (GJ-trp, GJ-tmp, and GJ-sbtrp). The South Asian subpopulation XI-2 is distinct from the other XI subpopulations. It has a genetic similarity to the cA group. Most of these results have already been described in studies of rice diversity (Wang et al. 2018a, b; Wang et al. 2018a, b). On the basis of the PCA, the Japonica accessions from Africa appear undifferentiated from those from Asia. They are fully encompassed in the Japonica cloud. The predominant Japonica subgroup in Africa is GJ-trp. There is only one GJ-tmp accession (from Tanzania) and one GJ-sbtrp accession (from Côte d'Ivoire) among 65 African GJ-trp accessions. Circum*-*Basmati rice (cB) and circum*-*Aus (cA) ecotypes are very rare in the African 3 K-RG sample. We identified only three cB accessions in Africa, two of which are in Madagascar, and one in Liberia, and only one cA accession, from Kenya. These four accessions do not show any particularity in the PCA space. In contrast, all Indica subgroups (XI-1A, XI-1B, XI-2, and XI-3) are found in Africa. Their distribution in the PCA space conforms to the Asian variation. Altogether there was no obvious differentiation of groups and subgroups from Africa compared to those from Asia.

The ADMIXTURE method served to test whether the African accessions exhibit specific ancestry composition compared to the major Asian groups (Fig. 1c). The cross-validation error is lowest for K = 9 (Fig. 1d). At K = 9, we find the same genetic groups that have previously been identified for this species. There was no cluster specific to African accessions, either Indica or Japonica, in this global inference analysis. The ADMIXTURE analysis assigned African accessions ancestral composition similar to those of Asian origin.

**Fig. 2:**
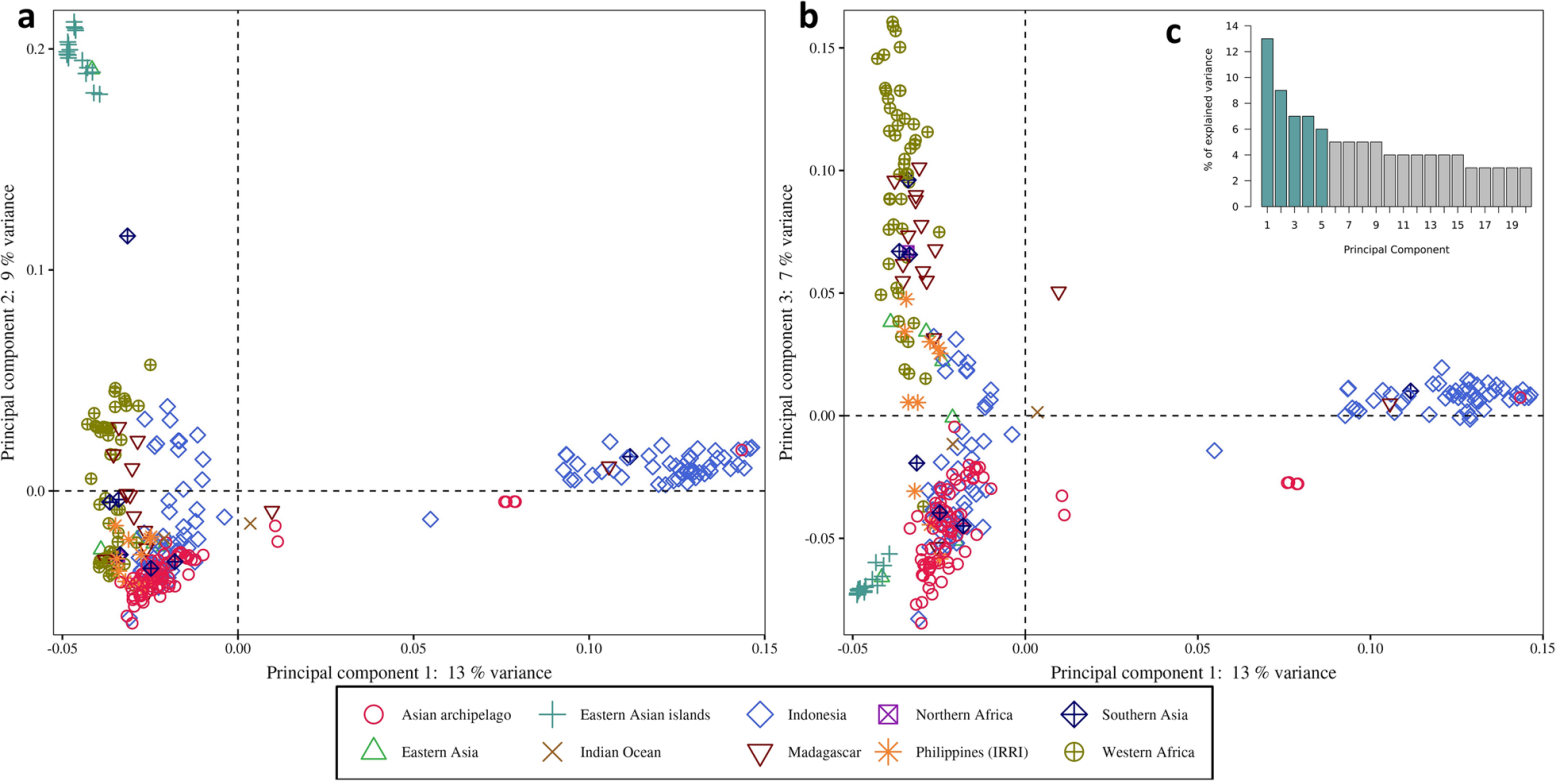
Principal Component Analysis on 292 Oryza sativa sub. japonica tropical accessions from Asia and Africa: This figure shows the dimensional structuring of GJ-trp based on their geographical origins (by shape and colour) on axes 1 and 2 (**a**), and on axes 1 and 3 (**b**). The first three dimensions explain 29% of the total variance. (**c**) Percentage of explained variance for the first ten principal components

PCA analyses on accessions representing only the GJ-trp reveal a slight geographical structuring of tropical Japonica groups (Fig. 2). The first axis (13% of variance) separates some of the GJ-trp from Indonesia from other Asian and African GJ-trp. The GJ-trp from the East Asian countries are distinguished from the other GJ-trp on the second axis (9% of variance). These results correlate well with the patterns observed by (Gutaker et al. 2020) within Japonica and more finely among varieties classified as tropical. On either side of axis 2, the West African GJ-trop (upland accessions) are divided into two groups (Fig. 2a). The West African GJ-trp stand out from most of the other GJ-trp on axis 3, together with accessions from Madagascar (Fig. 2b). As a result, axis 3, which accounts for 7% of the variance, allows Japonica accessions originating from different geographic areas to be distinguished, i.e. those from Africa versus Asia. At the interface, 24 Asian accessions are closer to the African group. Most of them are derived from breeding, such as “IR” materials from IRRI, except a set of ten landraces originating from Indonesia (Fig. 2).

### Local Ancestry Estimates

We investigated the genomic differentiation between African and Asian Japonica by chromosome painting with two approaches, PCA-KDE (Principal Components Analysis – Kernel Density Estimation) and ELAI (Efficient Local Ancestry Inference). PCA-KDE (Santos et al. 2019) was used to locally assign blocks of 150 SNPs to Japonica, Indica and cA references. The individuals selected to represent the ancestral poles faithfully reflect the perfect genetic diversity structure of the *Oryza sativa* species (Additional file 1: Fig. S1). For each chromosome, we represented the corresponding ideogram (Additional file 1: Fig. S2). Within chromosome 1, seven GJ-trp accessions from Asia (Additional file 1: Fig. S2) share an Indica/cAus haplotype of more than 10 Mb (from 9.2 Mb to 19.3 Mb). This haplotype block is not found in African GJ-trp (Additional file 1: Fig. S2).

**Fig. 3:**
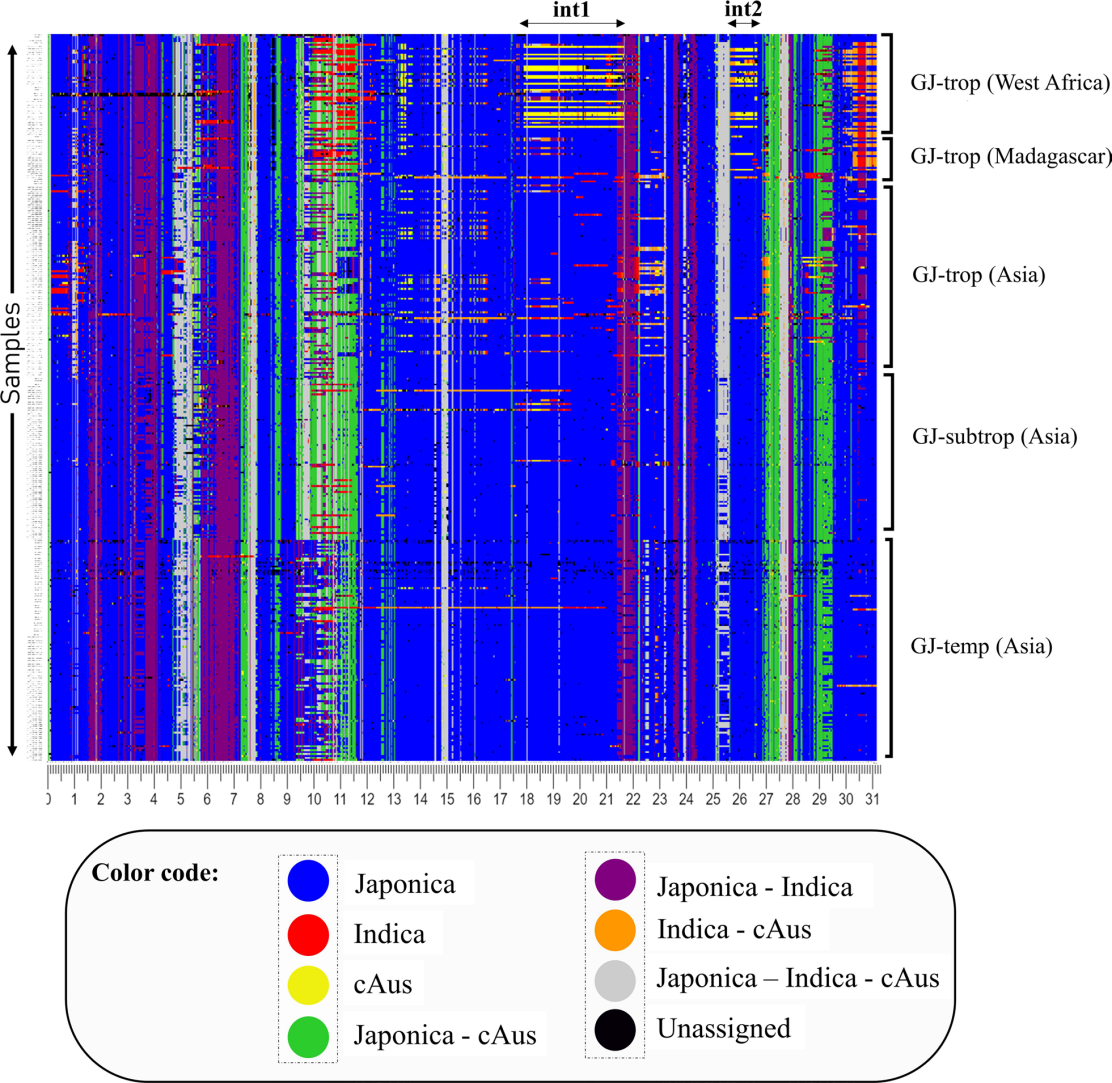
Local haplotype assignment on chromosome 6 of japonica varieties: Accessions are arranged from top to bottom by geographical subgroups. The base colours are defined according to whether a haplotype window has been assigned to one of these references: japonica (blue), indica (red) and cAus (yellow). However, when the haplotype is shared between two source populations, it is then coloured with the combined colors of its two main components: japonica-indica (purple), japonica-aus (green) and aus-indica (orange). When a haplotype is not group-specific, it is coloured grey. Finally, when the SNPs composing the haplotype window are absent from the source populations, it is shaded in black

A significant extended haplotype on chromosome 6 of 3.8 Mb (int1a) ranging from 17.9 to 21.7 Mb is shared by 14 accessions (Additional file 2: Table S4) of tropical Japonica (GJ-trp) from West Africa (Fig. 3). Two accessions (IRIS_313-11,103, IRIS_313-11,104) share a shorter cA haplotype (int1b, see Fig. 6c) between 17.9 and 20.26 Mb with the previous 14 accessions. The blocks assigned to cA are highlighted in Fig. 6c. We also note on the end of chromosome 6, between 25.8 and 26.7 Mb, a block of haplotype of cA ancestry on half of the GJ-trp accessions from Africa.

We have also identified a 600 Kb wide Indica introgression fragment at about 10.5 Mb of chromosome 6 that is specific to West African and Madagascar accessions. Our research (unpublished) shows that the segment introgressed by the tropical haplotypes of O. sativa ssp Japonica correlates most strongly with the haplotypes classified in the XI-2 group from O. sativa ssp Indica. This suggests that the introgressed segment may have been inherited from the Asian Indica XI-2 cluster, associated with a geographical origin in South Asia.

The findings of the ELAI allelic assay also highlight regions on chromosome 6 of cA ancestry in the GJ-trp rice population from Africa (Additional file 1: Fig. S3). Within the regions assigned to cA by the PCA-KDE method, ELAI consistently identifies an occurrence of cA alleles. ELAI differs from the PCA-KDE approach by directly estimating ancestry blocks by considering recombination through switch methods between different layers of the hidden Markov chain. The smoothness of the ideograms produced by the ELAI method is strongly influenced by the mg parameter (number of admixing generations). Therefore, this parameter affects the precision of allele inference in the source populations (Additional file 1: Fig. S4–S7). The results with mg = 20 are comparable with the PCA-KDE approach (Additional file 1: Fig. S8). The above results suggest a hybridization between the GJ-trp accessions and the circumAus subgroup in the generation of West African upland accessions.

### Testing for Introgression

**Fig. 4:**
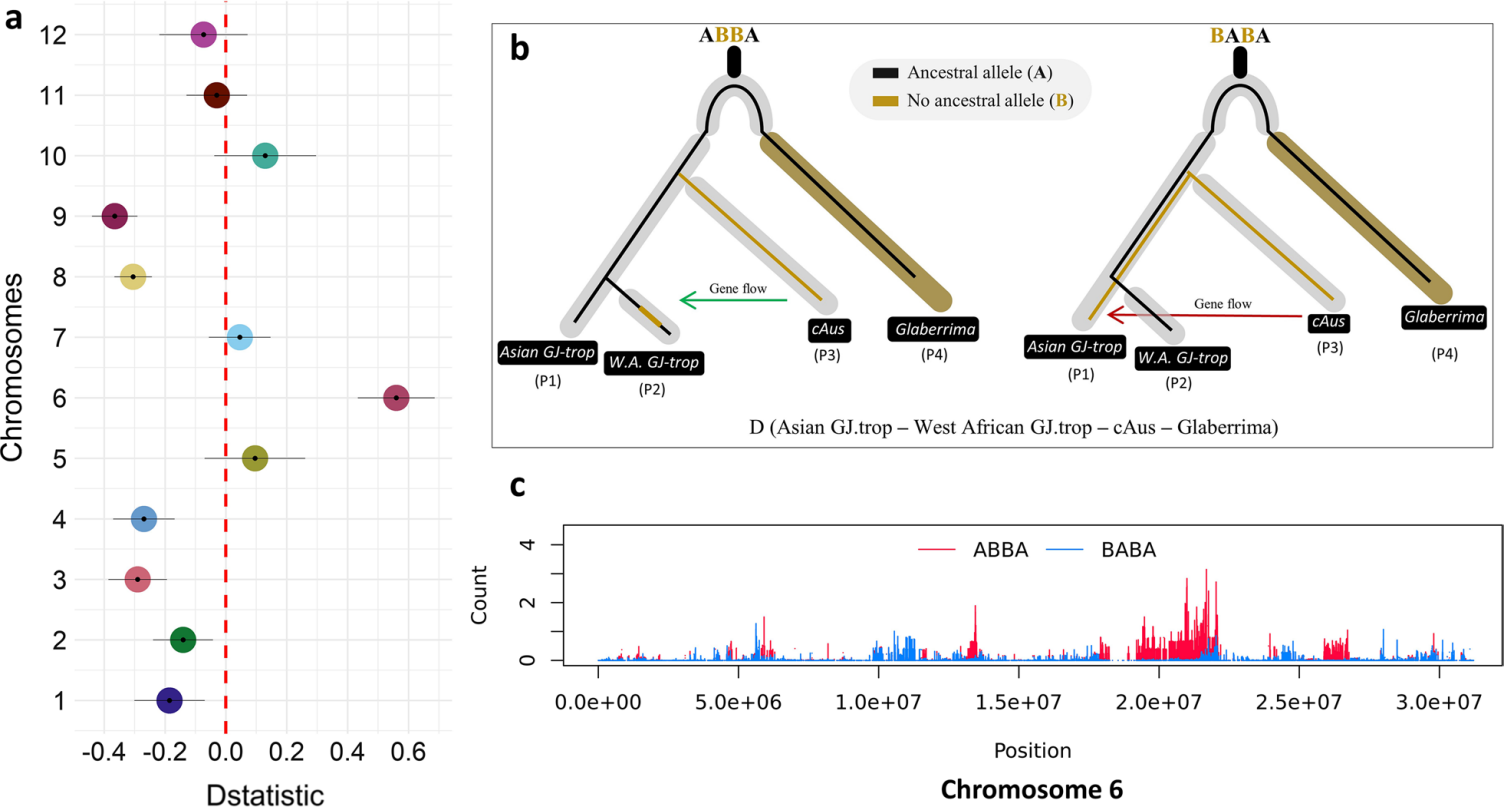
Hybridization between circum-Aus and African upland japonica varieties: **a** Dots are values of Patterson's D-statistic for each of the 12 chromosomes of the African upland rice varieties. The bars surrounding the points correspond to the standard deviation obtained with the Jackknife block procedure. A positive value of D indicates an excess of shared alleles between the P3 and P2 populations, while a negative value suggests an excess of shared alleles between P2 and P1. **b** A schematic illustration of Patterson's D test. The scenario proposed to test gene flow (excessive allele sharing) between circum-Aus accessions (P3) and African upland japonica varieties (P2), compared to Asian upland japonica rice (P1). The *Oryza glaberrima* species is used as an outgroup. The green arrow indicates gene flow from circum-Aus to African upland rice varieties, while red arrow indicate gene flow between cAus to Asian tropical japonica. **c** Patterns distributions of ABBA and BABA along chromosome 6 of African upland japonica rice accessions. This figure was obtained based on the scenario diagram in Fig. 5. The ABBA motif designates an excess of alleles from the donor circum-Aus (P3) in the recipient population (P2). The opposite BABA pattern designates an excess of alleles shared between the P1 and P2 populations. On the x-axis, the position in base pairs along chromosome 6, and on the y-axis the count of observed motifs

Local ancestry results identify at chromosome 6, a haplotype block of cA on sixteen GJ-trp accessions from West Africa. To assess whether this trace was left by hybridization or rather by incomplete lineage sorting (ILS), we applied the ABBA-BABA method (Durand et al. 2011; Green et al. 2010) on the whole genome, and per chromosome. The analysis was performed twice by randomly changing the cA accessions. The sizes of the respective populations P1, P2, P3 and P4 (Fig. 4b) used are 322, 50, 201 (two randomly selected sets) and 3, respectively. *O. glaberrima* was used as an outgroup (P4). The results of the D-statistics are indicative of gene flow between cA and GJ-trp from Africa. The significance of the D values was tested by computing the standard deviations (SD) with the Jackniffe block procedure. We subdivided the genome into 379 blocks. The genome-wide mean value is D = 0.039, which is not very far from D = 0 (null hypothesis). The associated Z-score value is 0.56, which means that D is not significantly different from 0 (D is significant when Z is less than -3 or greater than 3). As a result, we cannot conclude that gene flow occurred between cA and GJ-trp at the genome-wide level. We quantified the admixture proportion (f), from the P3 population (cA) to the P2 population (African GJ-trp accessions), following the procedure described by S. Martin(https://github.com/simonhmartin/tutorials/blob/master/ABBA_BABA_whole_genome/README.md). Consistent with the ideogram plots, the percentage of cA genome in the African GJ-trp found is 0.22%.

Looking at each chromosome independently (Fig. 4a) we detected a significantly positive D value only on chromosome 6 with an average D = 0.55 (z-score = 4.39) (Additional file 2: Table S2). This result suggests that the derived allele (alternative allele to the Nipponbare reference) is shared by the GJ-trp accessions from West Africa more often than expected by chance, and that the pattern of variation observed on chromosome 6 results from an introgression of cA origin into the West African GJ-trp accessions genetic background.

**Fig. 5:**
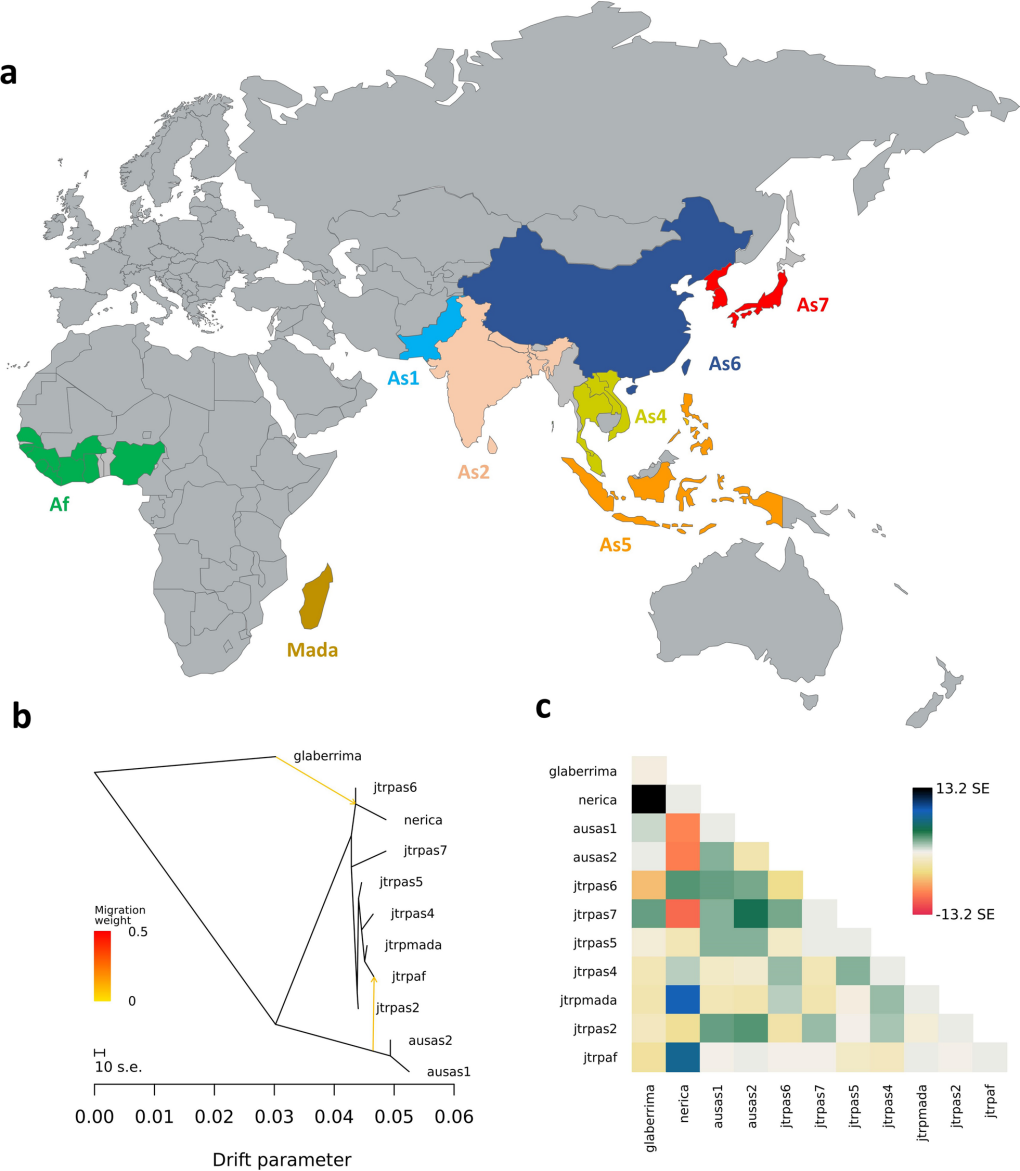
TreeMix analysis and geographical distribution of tropical japonica and cAus accessions: **a** Different geographical groups considered in the Treemix analysis. In Africa, tropical japonica accessions from the West African highlands and Madagascar. In Asia, from West to East, the main groups we considered are shown. The clusters are defined by geographical zone. Each coloured country holds at least one accession from the dataset used for the Treemix analysis. **b** Maximum likelihood tree inferred by Treemix in chromosome 6 for the different genetic (*Oryza sativa japonica*—*Oryza sativa aus*, *Oryza glaberrima*) and geographical (from Africa and Asia) subgroups. The yellow arrows between the taxa indicate gene flow events simulated by the model. **c** Residuals from the model used to obtain the tree

Using TreeMix, we were able to determine the genomic mixing patterns between cA and the geographically subdivided (Fig. 5a) tropical Japonica accessions (GJ-trp) between Asia and Africa (see Materials and Methods). The variance–covariance matrix shows that the model with no migration, m = 0, explains 95.3% of the total variance. In the absence of gene flow, this model predicts the most probable population splits. In the case where m = 1, the results show a first hybridization signal (in this case, the most relevant one) involving *O. glaberrima* species to generate the improved NERICA rice varieties. The inferred tree explains 98.3% of the variation with two simulated migration events (m = 2). Implemented on chromosome 6, we could again detect a signal of migration from cA accessions towards West African GJ-trp (Fig. 5b).

The Southeast Asian GJ-trp (As4 and As5) and West African GJ-trp are likewise genetically close. The TreeMix results show that the African GJ-trp accessions are more closely linked to the Southeast Asian GJ-trp, but also highlight a hybridization signal between the cA and the West African GJ-trp on chromosome 6, as suggested by Patterson's D statistic.

### Genetic Diversity Between Populations

The fixation index, abbreviated as Fst, assesses the degree of genetic differentiation between populations or distinct groups, ranging from 0 (no differentiation) to 1 (complete differentiation). The most striking global differentiation involves the GJ-trp As7 *vs* GJ-trp Int1 + with a Fst value of 0.62 (Additional file 1: Fig. S9). The Fst values are generally low among Asian populations. Fst is comparatively quite low between the tropical Japonica accessions of As2, As4, As5, and As6 (between minimum 0.01 and maximum 0.13) while it shows a slightly higher range value when As7 is involved (Fst increased from 0.19 for the lowest to 0.48 for the highest), confirming that tropical Japonica from North-East Asia (As7: Japan and Republic of Korea) appears to be distinct, possibly due to gene exchange with temperate forms. Looking at Fst values across the genome, we observe that the GJ-trp accessions from Madagascar appear to be genetically closer to the GJ-trp accessions from South Asia (As2), exhibiting a Fst of 0.02. But as can be seen (Additional file 1: Fig. S9), the upland GJ-trp accessions from West Africa that do not have the cAus introgression (Int1−) have a Fst value of 0.03 compared to the tropical accessions from Madagascar. When compared to West African upland accessions with the introgression (Int1 +), the Fst value is increased (Fst = 0.22).The measured difference between GJ-trp Int1 + and GJ-trp Int1− is 0.20.

**Fig. 6:**
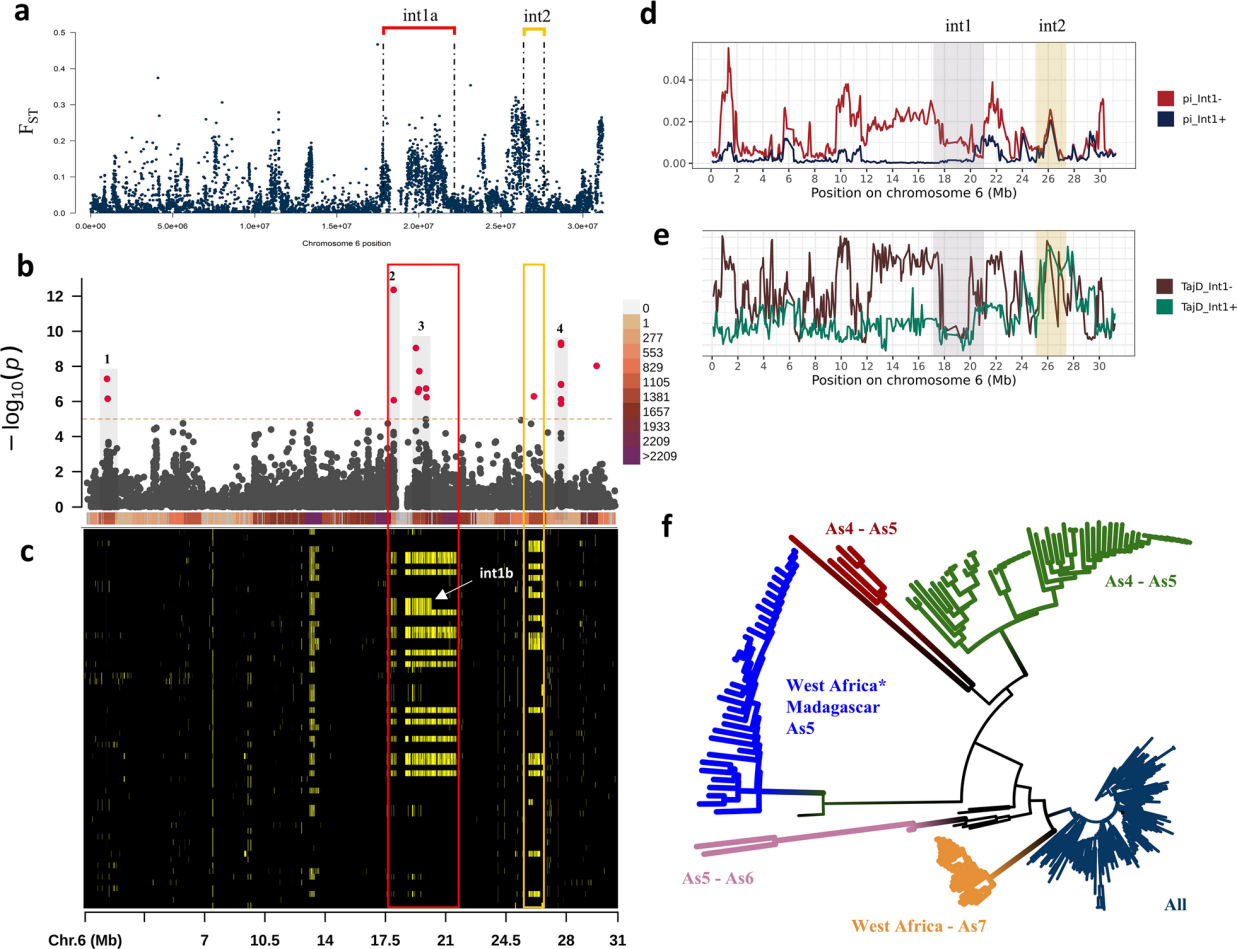
Genomic differentiation and population structure: **a** Fixation index (Fst) on chromosome 6 between Asian versus West African tropical upland Japonica rice populations. The position of the large introgression of cAus on this chromosome is represented by the segment in red. **b** Manhattan plot of the iHS statistic computed on the West African tropical upland Japonica rice population. The orange line corresponds to the significant threshold. The right legend shows the density of SNPs for each 1 MB window. **c** Highlight of the cAus genome introgression signal in the West African tropical Japonica genetic background on chromosome 6. The color in yellow identifies the chromosome locations with haplotypes assigned to cAus. **d** Nucleotide diversity (Pi) on chromosome 6 between tropical accessions with (Int + in blue) or without (Int- in red) the cAus introgression. The gray band identifies the region around the introgression. **e** Result of Tajima's D statistic on chromosome 6 between tropical accessions with (Int + in green) or without (Int- in brown) the cAus introgression. The gray band identifies the region around the introgression. **f** Unweighted neighbour tree constructed on the left outer edge of 100 Kb of the large introgression between 17.9 and 21.7 Mb on chromosome 6. West Africa annotated with an asterisk (*) denotes Tropical Japonica with cAus introgression

On chromosome 6, where we observed a signal of cAus introgression in the West African Tropical accessions, we found a very high value of Fst, differentiated between Int + and Int−. (Fig. 6a). The level of differentiation measured along chromosome 6 is quite similar between the West African tropical Japonica accessions with introgression and the other populations in Africa (tropical Japonica without introgression and the Madagascar accessions) and Asia (As2, As4, As5, As6 and As7). In all comparisons, a peak in Fst value was found on chromosome 6 between 18 and 22 Mb, corresponding to the region of cA introgression (Additional file 1: Fig. S10). The highest values were found between GJ-trop Int + *vs* GJ-trop As6 (0.8) or *vs* GJ-trop As7 (near 1). Unlike in comparisons of other Japonica groups without introgression, the Fst values are very heterogeneous, and the peaks appear to be located at the telomeric regions on q arm (Additional file 1: Fig. S11).

The level of intra-population diversity has no impact on Dxy, which measures the absolute genetic divergence between populations. It is more influenced by ancestral alleles and substitution rate. In contrast to the rest of chromosome 6, our results show a high level of absolute divergence around the introgression region of GJ-trop Int + relative to the different subgroups of tropical Japonica (Additional file 1: Fig. S12). This level of absolute divergence was not observed when we compared West African Japonica that did not introgress the cA fragment to Asian populations, nor when we compared Asian populations to Asian populations (Additional file 1: Fig. S13).

The degree of sequence polymorphism in a population is measured by nucleotide diversity (pi). The level of nucleotide diversity along chromosome 6 between tropical Japonica accessions from Africa shows a difference depending on whether the cAus introgression is present (Fig. 6d). Accessions with the cAus introgression (Int +) have a significantly lower level of genetic diversity than those without the introgression (Int−). GJ-trop As4 and GJ-trop Int + have remarkably low (near zero) pi values on chromosome 6 between 11 and 20 Mb (Additional file 1: Fig. S14). The GJ-trop Int + and GJ-trop AS7 populations have the lowest degrees of polymorphism, 0.0034 and 0.0087 respectively.

Tajima's D value, which detect non-random evolution of sequences, is overall negative for more than three quarters of the chromosome 6 in the African accessions with the cA introgression. This pattern was not observed in the other populations. Interestingly, African accessions that were not introgressed with cA have a high positive Tajima D value along chromosome 6, except between 18 and 20 Mb, where it falls below zero (Fig. 6e).

### Origin of the Introgression

We investigated the diversity in and around the main introgressions in order to derive information on the origin and the process that led to the observed patterns. We focussed on the main two cA introgressions in African Japonica accessions on chromosome 6.

For the larger one (Int1), we first analysed the first and last 100 kb segments (17.9–18.0 Mb 21.6–21.7 Mb, respectively) and verified that the clustering patterns assemble the Int1-bearing African upland accessions with cA accessions (Additional file 1: Fig. S15). We then extracted polymorphism data within the 3.8 Mb, between 17.9 and 21.7 Mb on chromosome 6 and compared the African upland GJ-trp that bear the introgression with the cA accessions. The African upland accessions that bear the full length Int1 all cluster on one clade in the resulting tree, whereas the two accessions with the smaller cA Int1 fragment (Additional file 1: Fig. S16) fall together further in the tree. The cluster of the full Int1-bearing accessions is homogenous and clearly separated within a large branch of cA accessions that displays hardly any structure. Altogether this suggests that Int1 has a single origin but that it does not point to a particular compartment of the cA group. For the smaller one (Int2), we extracted data for the 900 kb internal segment between 25.8 and 26.7 Mb and ran the same analysis. The global structure features three main branches corresponding to GJ, XI and cA groups. All the GJ-trp accessions that fell with the cA varieties, that is bearing Int2, formed a small cluster together with 14 cA varieties and three XI, three admixed and one cB varieties (Additional file 1: Fig. S17). Despite their low number, these accessions have very diverse geographic origins. Note that these 14 cA varieties are part of the large cA branch that bears the cluster of varieties which have Int1.

Regarding the location of “integration”, we looked at the most informative external borders, i.e. those that are shared by a majority of the introgression patterns and that fall in a region with an easily traceable origin. They were the left external border of Int1, that we characterized on the basis of the 17.7–17.8 Mb segment, and the right outer border of Int2, that we characterized with the 26.75–26.85 Mb segment. The other borders are less stable in location and fall into regions with complex origins, as illustrated by the intermediate colours on the ideograms of Fig. 3. The selected 100 kb regions are expected to be involved in the most recent introgression reduction.

The Int1 left outer border analysis reveals eight clearly distinct branches corresponding to cA, cB and several clusters within XI and within GJ. West African cultivars that have Int1 form a long branch with a sawtooth (annotated with asterisk in Fig. 6f) tip that connects close to the root of the tree, with varieties that do not have Int1 and originate from Africa, Madagascar and the Islands of Southeast Asia, Philippines and Indonesia (For more details see Additional file 1: Fig. S18a).

Given the broad diversity observed on this border, we extended the analysis to higher values along the chromosome and resolved the diversity patterns at the finest scale, enabling localization of the latest recombination point within less than a kb (between 17,904,819 and 17,905,605 see Additional file 2: Table S3).

For Int2, the analysis of the 100 kb between 26.75 and 26.85 Mb shows a classical pattern with Japonica, Indica and cA major branches bearing a limited number of exceptions. Japonica appears divided into several sub-branches and the West African Japonica varieties bearing Int2 are distributed in a GJ-trp sub-branch, in company with varieties from insular Southeast Asia, continental Africa and Madagascar (Additional file 1: Fig. S18b).

Considering the stability of the borders and the length of Int1 on chromosome 6 introgression, we attempted to extract within the Int1 region those loci which display specificity among the Int1 + materials in order to try and reconstitute the history of the introgression. The results are given in Additional file 2: Table S3. Two main cases were found: on one side 39 loci displayed an allele present in (almost) all Int1 + materials and absent or very rare in the Int1-; and on the other side, 6 loci displayed an allele found in only one Int1 + . No intermediate case was found, thus impeding meaningful phylogenetic analysis. The former 40 cases are helpful to trace the initial source materials. Most of the occurrence outside the Int1 + materials was concentrated in 13 accessions that have a broad geographic distribution, including outside Asia, and that are classified Indica, cA or admixed. It features multiple haplotypic combinations that suggest ancient populational relationships rather than recent cut and paste recombinational relationships. The latter 6 cases are likely to characterize recent mutations that occurred after the start of the introgression process. These data do not allow any clear or firm conclusions but they can be useful for further analyses with broader materials.

### Positive Selection of Candidate Region

Using the *rehh* v3.3.2 program (Gautier et al. 2017), we detected signals of selection on four regions on chromosome 6 in the African GJ-trp group. These locations are related with a list of fifteen outlier SNPs with p-values greater than the -log(*-p*-value = 5) threshold. The iHS statistic values for these fifteen SNPs are all negative, indicating that the alternative allele (non Nipponbare allele) is driving the selection. The candidate regions are shown in Fig. 6b. Two of these areas are situated between 17.9 and 21.7 Mb in the cA introgression region described above, while one region is located between 25.8 and 26.7 Mb in the second cA introgression region.

When performed on the Asian population, we detected signal of selection in two regions. They are between chr6:14,554,337 and 14,995,883 bp and chr6:21,497,039 and 21,702,660 bp (Additional file 1: Fig. S19). Positive selection would involve the reference allele (Nipponbare) with positive iHS values.

### Identification of Candidate Genes

**Fig. 7:**
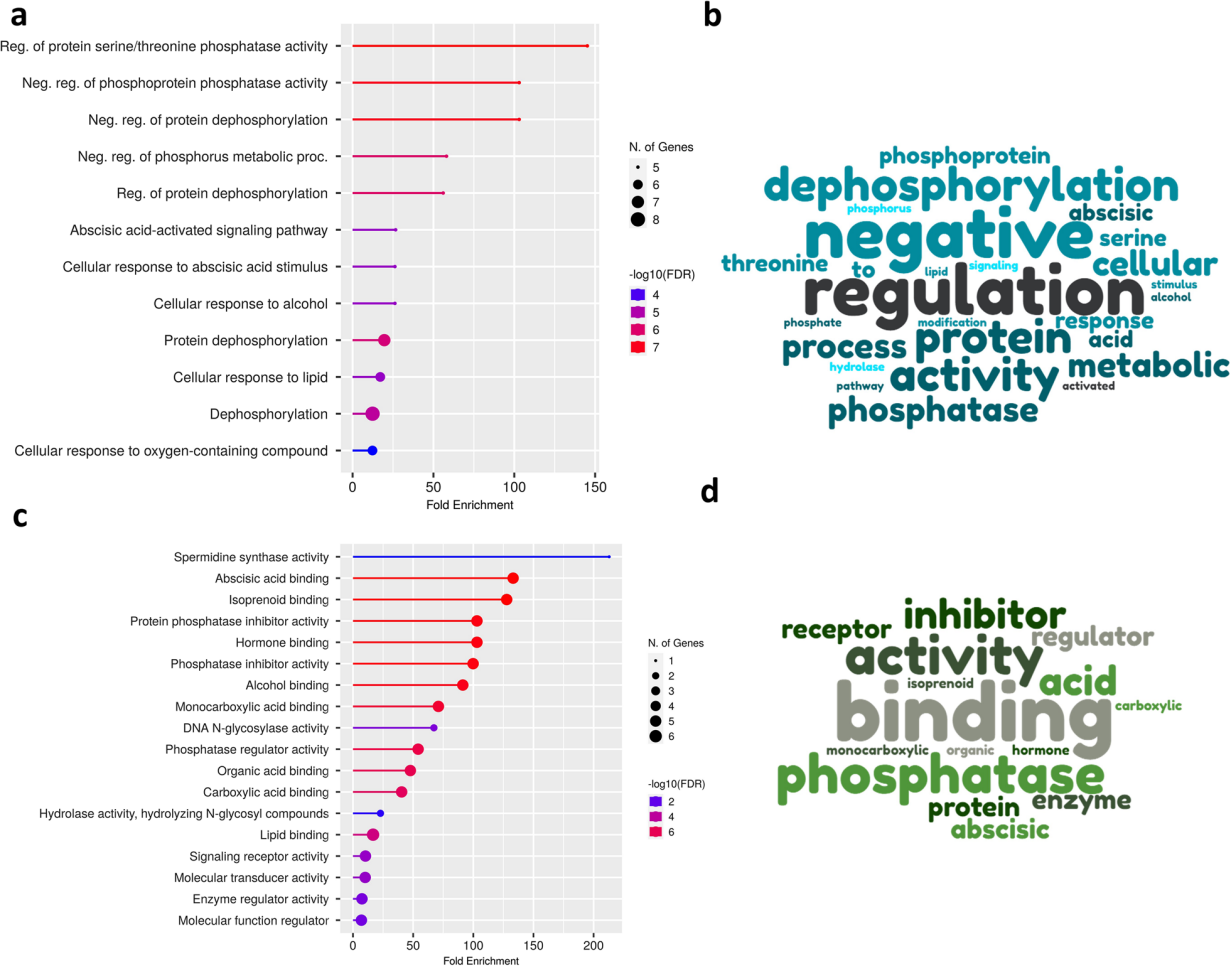
GO terms enrichment: Biological process **a** and molecular function **c** annotations of GO terms obtained from the http://bioinformatics.sdstate.edu/go database. The representation is limited to the most significant GO terms. Word cloud displaying the most frequent GO terms related to the respective categories of biological process **b** and molecular function **d**. The representativeness of a word is defined by its character size

The functions of the candidate genes have been studied in detail. We have discussed in some depth how these genes interact with the physiological processes of the plant. In a putative region with a substantial cA introgression (17.9—21.7 Mb) on chromosome 6, we found 46 non-redundant genes (Table 1). In the short cA introgression (25.8–26.7 Mb) of chromosome 6, twenty-one genes (Table 1) were discovered. Genes identified between 17.9 and 21.7 Mb are highly enriched in 43 biological processes (Additional file 2: Table S4), 28 molecular activities (Additional file 2: Table S5) and 4 cellular components (Additional file 2: Table S6). We only kept the GO terms annotations that had adjusted p-values below the cutoff of 0.01 in overall. Subsequently, twenty-three GO terms are evaluated, and they are divided into five key process categories (Additional file 2: Table S7). These terms, in turn, play important roles in: phosphatase activities, protein downregulation, abscisic acid (ABA) response pathway and cellular responses (Fig. 7a–b).

**Table 1:** List of candidate genes identified in the tropical West African japonica population between the two cA introgression regions

Gene	Name	Description
Os06g0504900	OsWRKY31	Similar to WRKY transcription factor 31
Os06g0507200	Prol-17	Bifunctional inhibitor/plant lipid transfer protein/seed storage domain containing protein
Os06g0507300	Os06g0507300	Similar to GAMYB-binding protein
Os06g0507400	Os06g0507400	Similar to GAMYB-binding protein (Fragment)
Os06g0507900	Os06g0507900	Conserved hypothetical protein
Os06g0508700	MetRS	Similar to methionyl-tRNA synthetase-like protein (ISS)
Os06g0517700	Os06g0517700	Similar to cDNA, clone: J075167K24, full insert sequence
Os06g0523900	Os06g0523900	RNA-processing protein, HAT helix domain containing protein
Os06g0524300	Os06g0524300	Protein of unknown function DUF3133 domain containing protein
Os06g0526100	OsbHLH054	Hypothetical conserved gene
None	None	None
Os06g0526350	Os06g0526350	Hypothetical conserved gene
Os06g0526400	OsPYL7	Similar to AT-rich element binding factor 3
Os06g0526466	OsEnS-89	Conserved hypothetical protein
Os06g0526600	OsABP	DEAD-box helicase ATP-binding protein, Response to abiotic stress (salt, dehydration, ABA, blue and red light
Os06g0526650	Os06g0526650	Non-protein coding transcript
Os06g0526700	OsPP2C55	Probable protein phosphatase 2C 55
Os06g0526800	OsPP2C56	Probable protein phosphatase 2C 56
Os06g0527100	OsCNGC12	Similar to Cyclic nucleotide-gated channel A (Fragment)
Os06g0527201	Os06g0527201	Hypothetical gene
None	None	None
Os06g0527300	OsCNGC13	Similar to cDNA clone:J023078M02, full insert sequence
Os06g0527500	Os06g0527500	Conserved hypothetical protein
Os06g0527800	OsPYL/RCAR7	Similar to Polyketide cyclase
Os06g0528300	OsPYL/RCAR8	Similar to Polyketide cyclase
Os06g0528600	OSSPDS2	Aminopropyl transferase
Os06g0532500	FCP2	Protein with similar CLE domain, Meristem maintenanc
Os06g0533700	Os06g0533700	Hypothetical conserved gene
Os06g0534200	Os06g0534200	Conserved hypothetical protein
None	None	None
Os06g0534500	Os06g0534500	Zinc finger, RING-type domain containing protein
None	None	None
None	None	None
Os06g0534800	Os06g0534800	Zinc finger, RING-type domain containing protein
None	None	None
Os06g0534900	Os06g0534900	Hypothetical conserved gene
Os06g0534950	Os06g0534950	Similar to zinc finger, C3HC4 type family protein
None	None	None
Os06g0547900	GSK4	Similar to Shaggy-related protein kinase eta (EC 2.7.1.-) (ASK-eta) (BRASSINOSTEROID-INSENSITIVE 2) (ULTRACURVATA1)
Os06g0551400	OsRab11	Ras-related small GTP-binding protein, Regulation of vesicular trafficking from trans-Golgi network to plasma membrane or vacuole, Jasmonic acid (JA)-mediated defense signalin
Os06g0553200	PFL	Similar to Meiosis 5
Os06g0556000	OsAAP12A	Amino acid permease, Transport of amino acid
Os06g0559400	Os06g0559400	Conserved hypothetical protein
Os06g0561000	OsMIOX	Myo-inositol oxygenase, Drought stress toleranc
Os06g0562200	OsPYL/RCAR9	Bet v I allergen family protein
Os06g0633300	OsPSK	Similar to Phytosulfokines 1
Os06g0633500	Os06g0633500	Zinc finger, RING/FYVE/PHD-type domain containing protein
Os06g0637500	R2R3-MYB	Similar to MYB transcription factor R2R3 type
Os06g0638000	Os06g0638000	Zinc finger, CCCH-type domain containing protein
Os06g0642900	Os06g0642900	Ubiquitin system component Cue domain containing protein
Os06g0642950	Os06g0642950	Non-protein coding transcript
Os06g0643000	Os06g0643000	Phox-like domain containing protein
Os06g0643050	Os06g0643050	Hypothetical protein
Os06g0643100	OsPBC1	Proteasome subunit beta type 3 (EC 3.4.25.1) (20S proteasome alpha subunit C) (20S proteasome subunit beta-3)
Os06g0643300	Os06g0643300	Development protein-like protein
Os06g0643500	Os06g0643500	Similar to ADR11 protein (Fragment)
Os06g0643600	MutM	DNA glycosylase/AP lyase domain containing protein
Os06g0643900	OsPAP26	Purple acid phosphatase (EC:3.1.3.2), Regulation of phosphate remobilization, Utilization of organic phosphoru
Os06g0644200	OVP1	Similar to Pyrophosphate-energized vacuolar membrane proton pump (Pyrophosphate-energized inorganic pyrophosphatase) (H + -PPase) (Vacuolar H + -pyrophosphatase)
Os06g0644800	OsOS-9	Glucosidase II beta subunit-like domain containing protein
Os06g0645700	OsHAF701	Similar to HAF1
Os06g0649000	WRKY28	PAMP (pathogen-associated molecular pattern)-responsive transrepressor, Defense respons
Os06g0649600	Os06g0649600	Non-protein coding transcript
Os06g0649800	Sdt97	Similar to DNA-3-methyladenine glycosylase I
Os06g0650300	OsglHAT1	Histone H4 acetyltransferase, Regulation of grain weight, yield, and plant biomas
Os06g0652000	OsRpo	Similar to T3/T7-like RNA polymerase (Fragment)

GO terms enrichment categorises candidate genes into two primary functional groupings (Additional file 2: Table S8). The first cluster includes 8 GO terms that are all involved in binding functions. The second cluster comprises genes with GO term enrichments involved in protein regulation/inhibition (Fig. 7c–d).

## Discussion

With this study, we made use of the reference dataset constituted by the 3 K rice genomes to investigate the expansion of *O. sativa* rice in Africa, with a focus on the GJ-trp subgroup of African upland rice, which are particularly well suited to drought-prone environments and are widely used as parents in breeding programmes (Saito et al. 2018).

Using a PCA analysis and the ADMIXTURE software to infer global population genetic structure, we recovered the well-known three-pole distinction between the Japonica, Indica, and cA. The African accessions show few representatives of the South Asian groups cA and cBas and an over-representation of the tropical forms of Japonica. Madagascar, as a potential bridge between Asia and Africa, has mainly Indica and Japonica varieties with, however, slight differences due to the absence of some of the Asian sub-groups (notably XI-1A, GJ-tmp and GJ-sbtrp) and to the existence of a specific local group, named Rojo, derived from Indica – Japonica—cA introgression, already documented (Ahmadi et al. 2021). At this scale of resolution, the variation in Africa seems affected by the course of the migration from Asia essentially through the loss of a few components which are, in Asia, confined to the Himalayan foothills and the northernmost part of the species distribution.

When focusing on the tropical Japonica group, we could reveal a slight differentiation between most accessions from Africa and most accessions from Asia. The bridge between these groups is made essentially by varieties from Indonesia and from the Philippines. The varieties from Madagascar are on the African side, reflecting the likely transfer pathway of upland rice from South-East Asia (Indonesia, Philippines) to Madagascar, and then from Madagascar to the African continent's interior as far as the western half of Africa.

Applying approaches to reveal introgressions that may have increased the variation of the Japonica group, we identified regions on chromosome 6 showing substantial contributions from cA ancestry in tropical Japonica from West Africa. One small region appears also in several varieties from Madagascar but the largest region, long of over close to 4 Mb, appears only in West Africa, in about 50% of the varieties we studied. Work conducted by McNally and colleagues (McNally et al. 2009) highlighted an introgression of cA/Indica on chromosome 6 of Moroberekan, a famous drought-tolerant (Acuña et al. 2008; Grondin et al. 2018) and blast-resistant (Carrillo et al. 2021) traditional Japonica rice variety. This introgression in Moroberekan matches the region we highlighted. This cultivar is native from West Africa, more specifically from Côte d’Ivoire. It is involved in several varietal breeding programmes (Girish et al. 2006; Grondin et al. 2018; Ishimaru et al. 2022; Khan et al. 2021; Kumar et al. 2020, 2014; Liu and Bennett 2011). The sixteen accessions showing this introgression, as well as Moroberekan, are localized among four bordering countries: Guinea, Sierra Leone, Liberia, and Côte d'Ivoire. Given its large size (3.8 Mb), it is likely a recent hybridization. However, the almost complete lack of other traces of the cA on the rest of the genome suggests a massive elimination of the cA genome, which can be expected to take many generations. The large size of the region can also be explained by the existence of several favourable genetic factors in it.

The global geographic pattern supports the idea that the initial hybridization could have occurred lately in West Africa because the introgression is not seen elsewhere. A recent study reports the occurrence of a cA landrace in a survey in Burkina Faso, as well as signs of introgression towards several other landraces, of the Indica group (Barro et al. 2021). Yet the scarcity of the cA group in Africa makes a post-introduction hybridization unlikely. Other counter-arguments are 1) the existence of another cA introgression on this chromosome, shared between West Africa and Madagascar, and the compatibility of the cA origin of both introgressions, making them possible derivatives of the same initial hybridization, and 2) the presence in Madagascar of the peculiar strain of Japonica which provided the left external border of the introgression. Note however that cA is not frequent in Madagascar either.

To determine whether this introgressions could be adaptive, we carried out genomic scans throughout the whole of chromosome 6 and we determined which locations are most likely to be subject to positive selection using the iHS statistic. The cA introgression regions on chromosome 6, which globally correspond to less than one fifth of the chromosome, harbour half of the 18 positive selection signals, including the strongest one. This result suggests that the introgression is adaptive. Given the recent occurrence of this introgression, we expect strong variation in linkage disequilibrium in the region of introgression. More analyses are needed to determine whether the pattern of LD due to the introgression itself could not lead to a signal of positive selection (Le Corre et al. 2020).

To go further into the potential role of the region of cA ancestry in the adaptation of Asian rice to Africa, we analysed the gene content of this region. Out of a total of 46 genes underlying this introgression, OsABP, OsCNGC, OsPP2C55, OsPP2C56, OsPYL/RCAR7, OsPYL/RCAR8, OsPYL7, and Prol-17 have functions directly related to responses to abiotic stresses such as salt stress and water stress (Bhatnagar et al. 2020; González-Guzmán et al. 2014; Han et al. 2019; Macovei et al. 2012; Macovei and Tuteja 2012; Min et al. 2020; Nawaz et al. 2014; Rodriguez 1998; Singh et al. 2014; Tian et al. 2015; Xu et al. 2015; Xue et al. 2008; Yadav et al. 2020; You et al. 2014).

The most significant GO terms are globally involved in regulatory functions that play a role in responses to abiotic stress. The main biological processes of these GO terms are phosphatase activity and dephosphorylation. In plants, the mechanisms that are triggered in the face of water stress are stomatal closure, ion transfers at the level of channels, and the activation of signalling pathways. Water stress affects the ability of plants to fix carbon dioxide (CO2), which in turn affects photosynthesis. Abscisic acid (ABA) is produced in significant quantities under water stress. ABA increases the production of ROS (reactive oxygen species), which increases the concentration of Ca2 + /Mg2 + cytosolic cations (Luan 2003; Murata et al. 2001). The candidate genes OSPP55 and OsPP56, which are members of the PP2C enzyme family and are regulated by Ca2 + and Mg2 + cations, are implicated in ABA signal transduction pathways (Luan 2003). OsCNGC, a CNGC (Cyclic nucleotide-gated channels) gene, is involved in pathogen defense and heat tolerance. Also related to Ca2 + signalling, a critical mechanism through which plants detect and react to stimuli (Nawaz et al. 2014). ABA stimulus responses are related to three terms, GO:0,009,738, GO:0,071,215, and GO:0,009,737. The OsPYLs (He et al. 2014) genes have been identified as the orthologs of the ABA receptors in rice. The presence of these ABA receptor genes (OsPYL/RCAR7, OsPYL/RCAR8, and OsPYL7) in the cA introgression suggests their function in water stress responses. Another possible adaptation gene is OsABP, which is regulated by abiotic stress. This gene is strongly affected by abiotic stresses such as salt, water stress, blue and red light, and ABA (Macovei et al. 2012). Further enrichment of GO terms for the molecular functions of these candidate genes is associated with abscisic acid binding functions and regulation of phosphatase activity. Under water stress, ABA binds to receptors (PYR/PYL/RCAR) and this complex then binds and inhibits the protein phosphatase enzyme PP2C (Daszkowska-Golec 2016). The guard cells control the stomata's closing by the inhibition of PP2C, which causes the phosphorylation of SnRK and then ABF, ABA-responsive element binding factors (Cutler et al. 2010; Ma et al. 2009; Min et al. 2019; Park et al. 2009). PYR—PYL—RCAR receptors play essential roles in drought tolerance (Muhammad Aslam et al. 2022) in rice (Kim et al. 2014). The significant enrichment of these GO terms points to a potential adaptive function for the candidate genes under abiotic stress. MAPK, which is involved in ABA signalling to regulate stomatal opening (Danquah et al. 2014; Liu et al. 2015), is assumed to play a significant role in the response to abiotic stress, particularly water stress.

The functions of candidate genes were thus identified as being strongly involved in the response to water stress using gene ontology (GO) enrichment and KEGG pathway annotation analyses. Meta-analysis identified two regions on chromosome 6 with high QTL abundance for root traits and drought avoidance (Khowaja et al. 2009). The root trait QTLs were discovered near the centromere, perfectly matching the positive selective scan signal on African upland. Drought avoidance QTLs were precisely located between 25,855,394 and 25,855,939 base pairs (Khowaja et al. 2009). This position overlaps with the second region of cA introgression (between 25.8 and 26.7 Mb) identified in West African upland accessions. However, our positive selection test analyses failed to detect a significant signal at this location. African upland rice varieties, exemplified by Moroberekan, are known for their remarkable drought tolerance, and several studies highlighted the high adaptability of the cA ecotype to high contrast environments due to drought or heat (Bernier et al. 2008; Casartelli et al. 2018; Dixit et al. 2012). This could indeed explain the advantage of this introgression in tropical West African Japonica.

We were able to pinpoint the reduction of genetic variation along migration of *O. sativa* rice from Asia, the origin of domestication, to the African continent and to confirm earlier assumptions about migration routes. However, we uncovered specific features of the genomic constitution of upland rice populations from West Africa, particularly an adaptive introgression signal derived from a cA contribution on chromosome 6, which bears genes potentially involved in drought responses. We illustrate how the evolution of genetic diversity along geographic migration can be used to circumvent the corpus of genes involved in crop adaptation. Introgressions leave traces which enable inventorying and specifying genomic localization, flow among ecotypes, phenotypic colocalization, all ingredients which will considerably support functional investigation in the future.

## Materials and Methods

### Genetic Material and Data Source

We used data from the 3 K-RG as a core dataset (The 3000 rice genomes project, 2014) available at https://snp-seek.irri.org/_download.zul. The metadata we used to match this dataset comes from (Wang et al. 2018a, b). The 3 K-RG were sampled from 89 countries, with 77.2% of the accessions originating from Africa and Asia (Fig. 1a). We selected the whole set of accessions available from Africa (*n* = 258), which were sampled in 25 countries. From Asia we only selected accessions representing traditional and promising material (based on metadata), i.e. we excluded improved varieties, which could have been influenced by the selection process like hybridization events between rice genetic groups. This resulted in a set of 2452 accessions originating from 21 Asian countries (Additional file 2: Table S9). The majority of East Asian countries and Asian islands are well represented in the sample beside the two major countries that are China and India. The majority of accessions representing West Africa are Japonica varieties, whereas, in East Africa, Indica varieties are more common. The Madagascar island is represented by a large number of accessions (*n* = 66), including both Japonica and Indica varieties.

In order to constitute an outgroup, we isolated three accessions whose name includes TOG, an abbreviation for *Tropical Oryza glaberrima* which suggests that they belong to the species *O. glaberrima*. Two supplementary accessions were added to these three accessions, accession Malogbana (from Côte d'Ivoire) and accession Kaushi (from Nigeria), which appeared highly distant from *O. sativa* accessions and similar to TOG varieties on the basis of a preliminary analysis we did using all the 3023 samples accessible through the 3 K-RG.

Along the same line, we selected four improved varieties, NERICA 1, NERICA 2, NERICA 8, and NERICA 9, expected to be hybrid derivatives between an *O. sativa* Japonica and *O. glaberrima* (Saito et al. 2018), to serve as a control for gene flow inference in our analyses.

This 3 K-RG sequencing project offered over 29 million bi-allelic markers in alignment with the *Nipponbare* reference genome. The genome sequences average 16X and 29X sequencing depth for African and Asian accessions, respectively. For our dataset (*n* = 2710), the mean coverage for accessions sampled in Africa is 90% compared to 93% for Asian accessions. The sequencing data, therefore, have a high quality according to these two parameters. A total of 10,459,872 bi-allelic SNPs was obtained after removing loci with more than 1% missing data. SNP markers are overall well distributed throughout the 12 chromosomes (Additional file 1: Fig. S20).

## Methods

### PCA, and Model-Based Clustering

Principal component analysis (PCA) was initially used to study the relationship between African and Asian *O. sativa* accessions. PCA was implemented using PLINK v1.90 (Purcell et al. 2007). We used 7,315,477 unlinked SNPs after filtering on linkage disequilibrium (LD) with plink and the parameter *–indep-pairwise 50 10 0.2*.

We used ADMIXTURE (Alexander et al. 2009), in order to infer ancestral populations based on a maximum likelihood approach. We ran ADMIXTURE on the set of 2710 individuals described above and reduced the set of 74,373 markers, selected after an LD-pruning (*–indep-pairwise 50 10 0.2*) with a missing data filter (*–geno 0*) using PLINK. We ran ADMIXTURE for K, the number of ancestral populations, varying between K = 2 to K = 10. Analyses were performed with a random seed changing over time. The number of clusters K with the lowest cross-validation error was determined to be optimal.

### Local Ancestry Inference

We used two main approaches to infer local ancestry along the genome. The first is a method based on Kernel Density Estimation in PCA feature space (PCA-KDE) (Santos et al. 2019). The second method is ELAI (Efficient Local Ancestry Inference) (Guan 2014).

### Kernel Density Estimation in PCA Feature Space

PCA was used on windows of 150 SNPs for overall accessions of the 3 K-RGs and with more than 10 M SNPs. We overlapped each new window with half of the previous window for more accurate dimension reduction results by PCA (75 SNPs). We performed 139,464 PCAs on the complete genome. We estimated the kernel density of the five initial main components of each PCA. The Parzen-Rosenblatt window method is a kernel density estimator that uses a mixture of kernels to approximate a data distribution. It is a common non-parametric method for estimating a probability density function that does not require any prior knowledge or assumptions about the underlying distribution. The KDE computes the likelihood of each haplotype being allocated to one of the three reference groups in a window of 150 SNPs, and then for all windows.

### Efficient Local Ancestry Inference (ELAI)

In order to constitute reference materials for the three ancestral poles Japonica, Indica and cA, we chose varieties that appeared among the purest based on ADMIXTURE. Each accession was chosen with an arbitrary cut-off of 80% pure ancestry. Any unclassified accession based on these criteria is considered an admix. The distribution of the reference accessions on a PCA illustrates their distinctiveness and intra-group purity Additional file 1: (Fig. S1).

ELAI was directly applied to our diploid data. The trained population included 2418 individuals, while the source populations included 130, 97, and 65 accessions for Japonica, Indica, and circum-Aus, respectively.

As recommended by the author (Guan 2014), we defined 03 upper-layer clusters corresponding to the source populations, and 15 Lower layers clusters (5 times the number of upper clusters) and we performed ELAI with several values of the parameter mg (20; 50; 100 and 1000), which corresponds to the number of generations after hybridization. All runs were done with 30 steps of Expectation–Maximization (EM), an iterative algorithmic approach to finding the maximum likelihood of the parameters in the statistical models. All loci with a missing data rate of more than 0.05 and a minor allele frequency less than 0.01 were excluded from the analysis.

### Detection and Origin of Introgression

The ABBA-BABA approach was used to determine if signals of introgression between *O. sativa* subspecies are attributable to hybridization or due to an incomplete lineage sorting (ILS). Considering A as the ancestral alleles and B as the derived alleles, the method compares the number of loci in a particular genome that correlates to ABBA or BABA genotypic patterns in several populations P1, P2, P3, and P4. P1 and P2 are closely related taxa, P3 is a near phylogenetic taxon to P1 and P2, while P4 is a distant one (outgroup). Under incomplete lineage sorting (no gene flow), P1 and P2 should have roughly the same proportion of P3 alleles. This indicates that there would be no difference in the quantity of ABBA and BABA motifs. However, if hybridization results in introgression from the donor taxon P3 to a recipient taxon P1 or P2, P3 may share more alleles with either taxon. (Green et al. 2010). The D statistic may be defined as follows:

$$\user2{ D } = \left[ {\user2{sum }\left( {{\varvec{ABBA}}} \right) - \user2{ sum }\left( {{\varvec{BABA}}} \right)} \right]\user2{ }/\user2{ sum }\left( {\user2{ABBA } + \user2{ BABA}} \right)$$

As a result, a positive value of D indicates that there are more ABBA motifs than BABA motifs and that gene flow has occurred from P3 to P2. When D is negative, the BABA pattern is more prominent, corresponding to a greater allele exchange between P3 and P1. The null hypothesis of the statistic (D = 0) might be read as no gene flow between taxa.

The D-statistics were calculated using genotypic data from tropical Japonica accessions from Asia and West Africa as P1 and P2, respectively. The Asian circum-Aus varietal group was selected as the donor population (P3). The species *O. glaberrima* was assumed to be the outgroup (P4). The study was repeated twice, with varying numbers of individuals in each taxon. The same number of markers (10,459,872 SNPs) were used in both analyses. The Jack-knife block approach was used to i) determine the standard deviation and variance of the D-statistic values and ii) rectify the correlations between loci caused by linkage disequilibrium. We applied Simon H. Martin script (https://github.com/simonhmartin/genomics_general).

We investigated where the introgression originated and where it was integrated. Using diversity trees created both inside the introgressions (in contrast to cA) and outside (in comparison with Japonica). To construct the trees, we used the NJ function of the APE package (Paradis et al. 2004) which is based on neighbour-joining tree estimation of Saitou and Nei (1987) (Saitou and Nei 1987).

### Phylogenetic Analysis

We used TreeMix (Pickrell and Pritchard 2012) to investigate phylogenetic relationships and population splitting patterns between tropical Japonica accessions from Africa and those from Asia. TreeMix builds on a maximum likelihood genetic drift tree to infer relationships between groups using maximum likelihood trees and considering both splits and potential gene flow. We ran TreeMix on the allele frequency data of tropical Japonica varieties (from Africa and Asia), on Asian cA and on the species *O. glaberrima*, which was used to root the tree. Ten iterations were made assuming between 0 and 10 possible migration edges. Standard errors were estimated in blocks of 500 SNPs. Each run was done with a random seed and a bootstrap.

This analysis was done at the genome and chromosome level. We created clusters that grouped accessions into more specific populations based on geography. We defined three groups for accessions from Africa: upland varieties from West Africa, GJ-trp accessions from Madagascar and NERICA varieties of interspecific origin. Asian GJ-trp and cA accessions were separated into seven groups on a geographical basis, as in Fig. 5a.

### Signatures of Positive Selection

To determine whether introgression patterns could be associated with adaptive processes, we looked for footprint of selection. We used the standardized intra-population integrated haplotype score (iHS), based on the Extended Haplotype Homozygosity (EHH) (Gautier et al. 2017). iHS detects a partial selective sweep in a single population. iHS is calculated from the EHH values, which estimate the probability that two randomly selected haplotypes are identical up to a distance x around a focal marker (Voight et al. 2006). Thus, for biallelic data, at a focal locus, the EHH statistic compares the range of haplotypes carrying either the ancestral or the derived allele.

Genomic scanning for traces of selective sweeps was done in each of the West African and Asian tropical Japonica rice populations. We focused only on chromosome 6. Polarization is necessary to define which of the two alleles is ancestral or derived. We chose not to polarise the data and instead considered the reference allele versus the alternative allele. Data were phased using the software BEAGLE V5.2 (Browning and Browning 2007) using default settings.

For a given reference allele, the integrated EHH (iHH) is defined as the area under the EHH curve, which in turn is defined by the EHH values and the chromosomal positions associated with each allele iHH_ref and iHH_alt (Voight et al. 2006). iHS, defined as ln(iHH_ref/iHH_alt), is constructed to have an approximately standard Gaussian distribution, then the transformation of the iHS values into p-values is done according to the following equation:

$${\varvec{p}}\left( {{\varvec{iHS}}} \right)\user2{ } = \user2{ } - \user2{ log}10\user2{ }\left[ {1 - 2\user2{ }\left| {\user2{ Phi }\left( {{\varvec{iHS}}} \right) - 0.5} \right|} \right]$$

where Phi(x) is the distribution of the Gaussian cumulative distribution function.

### Genetic Diversity Analysis

In a pairwise comparison between subgroups, we computed the genome-wide fixation index (Fst) for each of the defined GJ-trp subgroups. We used the same set of markers as the Admixture analysis (74,373 SNPs). We used the compute *pairwise.fst.dosage* function from the hierfstat package (Weir and Goudet 2017).

We considered the same groups as in the TreeMix analysis, excluding the NERICA improved varieties, to calculate the diversity statistics along the chromosome. Because the Pi, D_xy_, and Tajima's D statistics are influenced by the number of samples, we have adjusted the group sizes as a precaution. Each group has roughly the same number of samples (15 or 16), with the exception of two groups, the accessions from Asia 2 (India and Sri Lanka) and Asia 6 (China and Taiwan), which have 5 and 8 accessions, respectively. There are 171 accessions in the Asia 5 group. To avoid any bias, we randomly selected 15 accessions from the Philippines (GJ-trp_As5phi) and 15 accessions from Indonesia (GJ-trp_As5ind) from the traditional types. The West African accession populations were divided into two smaller groups as well. GJ-trp Int^+^ and GJ-trp Int- represent upland Japonica rice with and without the cA introgression on chromosome 6. This distinction will facilitate assessing the impact of the introgression on genetic diversity.

On 100 kb sliding windows that overlapped by 50 kb at each step, standard molecular diversity statistics such as nucleotide diversity (Pi), average number of nucleotide substitutions per site (D_xy_), neutrality test such as Tajima's D, and fixation index (Fst) were calculated for each chromosome. All sites with fewer than ten SNPs were excluded from the analysis. We used the popgenWindows.py function from Simon Martin's general genomics repository on GitHub.

### Candidate Gene Identification and Characterization

We applied a custom script to detect all underlying genes in the 30 kb of significant SNP markers in the iHS data with a p-value greater than 5. The gene list utilised was based on the 3 K-RG vcf annotation file, which can be found at https://snp-seek.irri.org, and the funRiceGenes database, which contains over 4100 functionally defined rice genes and over 6100 gene family members (Huang et al. 2022).

We used Gene Ontology enrichment (GO) and the Kyoto Encyclopedia of Genes and Genomes (KEGG) annotation analysis to characterise the function of each candidate gene. The annotations of the list of identified genes were made on three different databases, namely:https://shinyapps.southgreen.fr/app_direct/goenrich/, http://bioinformatics.sdstate.edu/go/, and http://geneontology.org/. All annotations were based on genes from the *O. sativa Japonica* group. The gene ontology platform http://geneontology.org/ contains 35,775 annotated genes, http://bioinformatics.sdstate.edu/go/ 43,658 annotated genes. The SouthGreen database (https://shinyapps.southgreen.fr/app_direct/goenrich/) is contains 23,733 and has roughly 910 K GO terms. All significant signalling pathways with an FDR less than 0.05 were kept.

The nominal P-value from the hypergeometric test is used to calculate FDR. Fold Enrichment is defined as the percentage of genes in our list that belong to a pathway divided by the corresponding percentage in the background. FDR reveals the probability of enrichment by chance. Fold Enrichment, as a measure of effect size, indicates how significantly genes from a specific pathway are overrepresented (for more details http://bioinformatics.sdstate.edu/go/).

## Supplementary Information

**Additional file 1 Figure S1**: Principal Component Analysis using references samples. **Figure S2**: Chromosome painting using PCA-KDE for 12 chromosomes. **Figure S3**: ELAI result in West African Upland on chromosome 6. **Figure S4**: ELAI result in chromosome 6 with parameter mg=20. **Figure S5**: ELAI result in chromosome 6 with parameter mg=50. **Figure S6**: ELAI result in chromosome 6 with parameter mg=100. **Figure S7**: ELAI result in chromosome 6 with parameter mg=1000. **Figure S8**: Comparison between PCA-KDE and ELAI results. **Figure S9**: Pairwise Fst comparison between intercontinental Tropical japonica populations. **Figure S10**: Pairwise Fst comparison between GJ-trop Int+ *vs *others subgroups of GJ-trop. **Figure S11**: Pairwise Fst comparison between tropical japonica populations. **Figure S12**: Pairwise Dxy comparison between GJ-trop Int+ *vs *others subgroups of GJ-trop. **Figure S13**: Pairwise Dxy comparison between tropical japonica populations. **Figure S14**: Pairwise Pi comparison between tropical japonica in chromosome 6. **Figure S15**: Trees constructed on 100 Kb internal to the right and left extremities of the Int1 introgression. **Figure S16**: Unweighted NJ tree between cAus and West African tropical japonica rice. **Figure S17**: Unweighted NJ tree using 900 Kb internal segment between 25.8 and 26.7 Mb (Int2). Figure S18: Trees built on 100 Kb of the left and right borders of the Int1 and Int2 introgressions respectively. **Figure S19**: iHS statistic result in Asian tropical japonica. **Figure S20**: SNPs density along 12 chromosomes

**Additional file 2. Table S1:** The most significant biological process enrichments considered in this study. Retrieved from https://shinyapps.southgreen.fr/app_direct/goenrich/. **Table S2:** 291 Tropical japonica accessions from both Africa and Asia. **Table S3:** Detailed analysis of the cAus introgression on chromosome 6, located between 17.9 and 21.7 Mb. **Table S4:** Tropical japonica accessions having Int1 (Int1a and Int1b) cAus introgression on chromosome 6 are listed below. **Table S5:** Results of Patterson’s D statistic using ABBA-BABA tests. **Table S6:** iHS genomic scan results on chromosome 6 of the West African tropical japonica population. **Table S7:** Tropical japonica, cAus, NERICA and glaberrima accessions groups used for TreeMix analysis. **Table S8:** Results of Patterson’s D, fd and fdM statistic using ABBA-BABA tests on chromosome 6. **Table S9:** 2710 accessions samples from Africa and Asia. This table was adapted from the additional data in the article Wang and al. 2018: https://www.nature.com/articles/s41586-018-0063-9.

## Data Availability

All data used in this paper are available at https://snp-seek.irri.org/
