# Supplementary material for: Traces of Introgression from cAus into Tropical Japonica Observed in African Upland Rice Varieties
Source: Rice (N Y). 2023 Feb 28;16:12. doi: 10.1186/s12284-023-00625-4 (PMC9975138; doi:10.1186/s12284-023-00625-4)
Supplement: Supplementary file 1 — Additional file 1 Figure S1: Principal Component Analysis using references samples. Figure S2: Chromosome painting using PCA-KDE for 12 chromosomes. Figure S3: ELAI result in West African Upland on chromosome 6. Figure S4: ELAI result in chromosome 6 with parameter mg=20. Figure S5: ELAI result in chromosome 6 with parameter mg=50. Figure S6: ELAI result in chromosome 6 with parameter mg=100. Figure S7: ELAI result in chromosome 6 with parameter mg=1000. Figure S8: Comparison between PCA-KDE and ELAI results. Figure S9: Pairwise Fst comparison between intercontinental Tropical japonica populations. Figure S10: Pairwise Fst comparison between GJ-trop Int+ vs others subgroups of GJ-trop. Figure S11: Pairwise Fst comparison between tropical japonica populations. Figure S12: Pairwise Dxy comparison between GJ-trop Int+ vs others subgroups of GJ-trop. Figure S13: Pairwise Dxy comparison between tropical japonica populations. Figure S14: Pairwise Pi comparison between tropical japonica in chromosome 6. Figure S15: Trees constructed on 100 Kb internal to the right and left extremities of the Int1 introgression. Figure S16: Unweighted NJ tree between cAus and West African tropical japonica rice. Figure S17: Unweighted NJ tree using 900 Kb internal segment between 25.8 and 26.7 Mb (Int2). Figure S18: Trees built on 100 Kb of the left and right borders of the Int1 and Int2 introgressions respectively. Figure S19: iHS statistic result in Asian tropical japonica. Figure S20: SNPs density along 12 chromosomes [file 12284_2023_625_MOESM1_ESM.pdf]

Traces of introgression from cAus into tropical Japonica observed in African upland rice varieties

Abdoulaye Beye [a,b,d], Claire Billot [a,b], Joëlle Ronfort [b], Kenneth L. McNally [c], Diaga Diouf [d], and Jean-Christophe Glaszmann [a,b]

[a]CIRAD, UMR AGAP Institut, F-34398 Montpellier, France
[b]UMR AGAP Institut, Université de Montpellier, CIRAD, INRAE, Institut Agro, F-34398 Montpellier, France
[c]International Rice Research Institute, DAPO Box 7777, Metro Manila 1301, The Philippines
[d]Laboratoire Campus de Biotechnologies Végétales, Département de Biologie Végétale, Faculté des Sciences et Techniques, Université Cheikh Anta Diop, Code postal 10700, Dakar-Fann, Dakar, Sénégal
[*]glaszmann@cirad.fr

Supplementary Figures

Figure S1: Principal Component Analysis using references samples

Figure S2: Chromosome painting using PCA-KDE for 12 chromosomes

Figure S3: ELAI result in West African Upland on chromosome 6

Figure S4: ELAI result in chromosome 6 with parameter mg=20

Figure S5: ELAI result in chromosome 6 with parameter mg=50

Figure S6: ELAI result in chromosome 6 with parameter mg=100

Figure S7: ELAI result in chromosome 6 with parameter mg=1000

Figure S8: Comparison between PCA-KDE and ELAI results

Figure S9: Pairwise Fst comparison between intercontinental Tropical japonica populations

Figure S10: Pairwise Fst comparison between GJ-trop Int+ *vs* others subgroups of GJ-trop

Figure S11: Pairwise Fst comparison between tropical japonica populations

Figure S12: Pairwise Dxy comparison between GJ-trop Int+ *vs* others subgroups of GJ-trop

Figure S13: Pairwise Dxy comparison between tropical japonica populations

Figure S14: Pairwise Pi comparison between tropical japonica in chromosome 6

Figure S15: Trees constructed on 100 Kb internal to the right and left extremities of the Int1 introgression.

Figure S16: Unweighted NJ tree between cAus and West African tropical japonica rice

Figure S17: Unweighted NJ tree using 900 Kb internal segment between 25.8 and 26.7 Mb (Int2)

Figure S18: Trees built on 100 Kb of the left and right borders of the Int1 and Int2 introgressions respectively.

Figure S19: iHS statistic result in Asian tropical japonica

Figure S20: SNPs density along 12 chromosomes

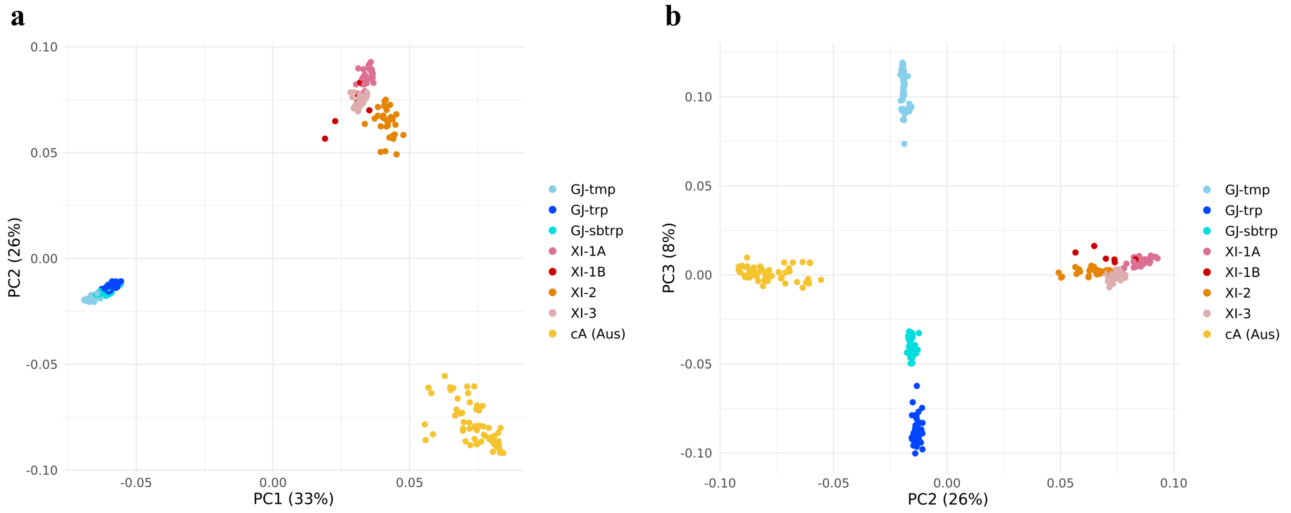

Figure S1. PCA on pure accessions selected as representing ancestral poles. These individuals were used for local inference analyses. There were 130, 97, and 65 accessions for japonica, indica, and circum-aus, respectively.

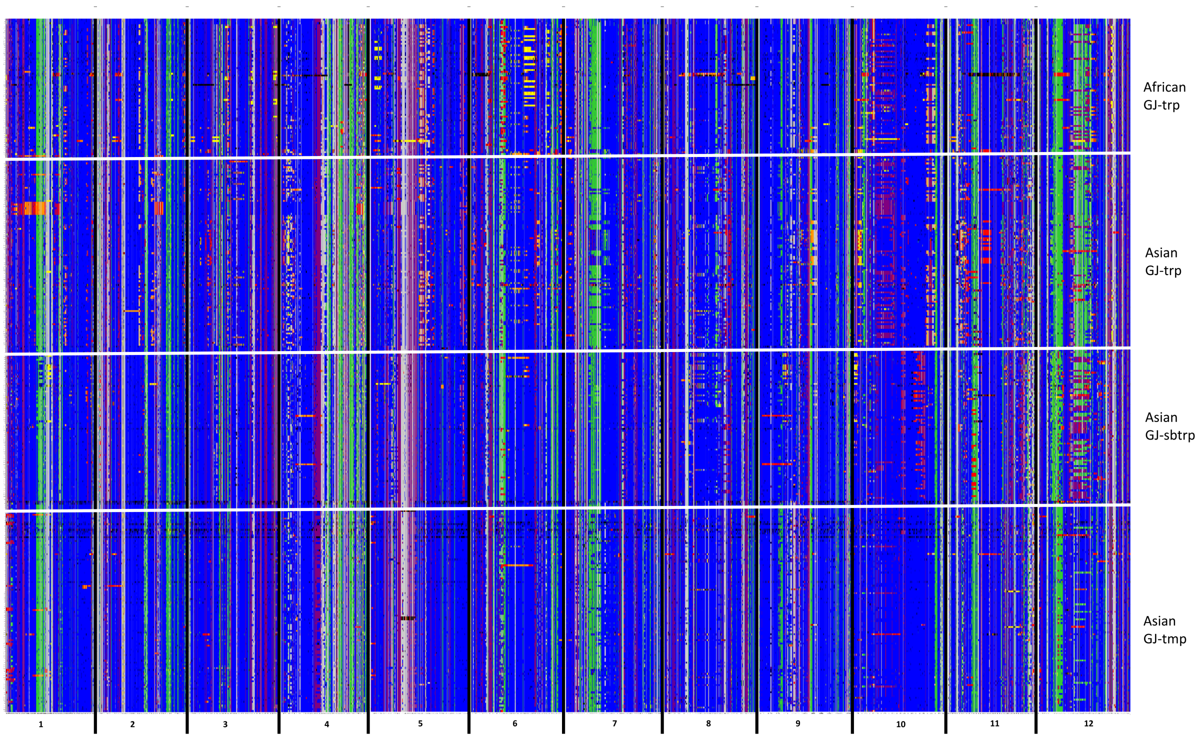

Figure S2. Chromosome painting for 12 chromosomes of Oryza sativa japonica group using PCA-KDE approach. This analysis was restricted to japonica. The japonica genome is represented by the blue colour. The cAus and Indica subgroups are represented by the colours yellow and red, respectively. The accessions are grouped into rows in the following order: Tropical japonica from West Africa - tropical japonica from Asia - subtropical japonica from Asia - temperate japonica from Asia.

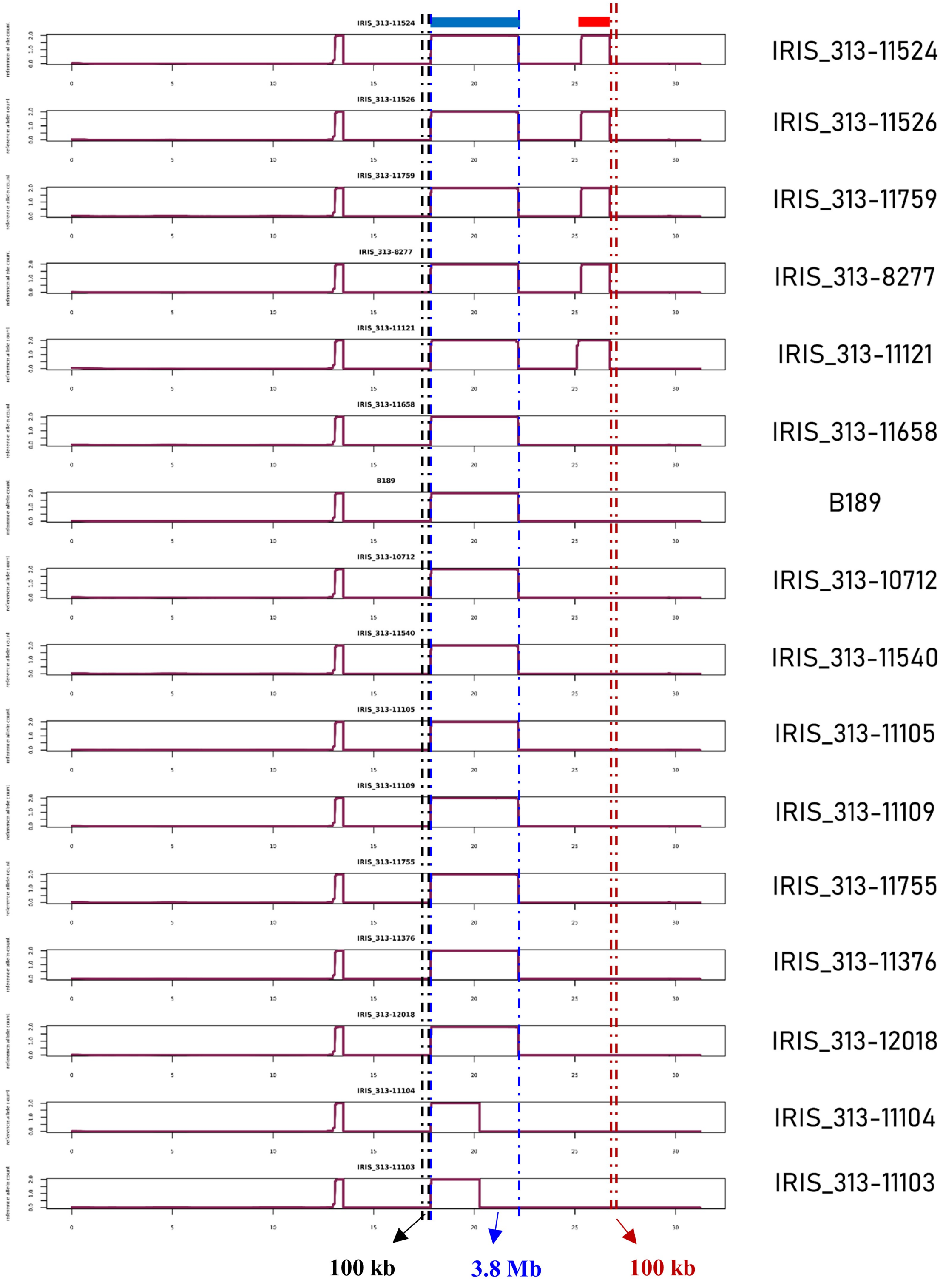

Figure S3. ELAI result in chromosome 6 for West African upland rice. List of 16 African tropical Japonica accessions that have been identified as having cAus introgression on chromosome 6. The purple curve depicts the cAus allelic dosage on chromosome 6. The blue and red markings highlight places where both alleles of cAus are found. The black dashes delineate the 100 kb of chromosome 6 extracted to study the integration site of introgression. The darkblue dashes designate the 3.8 Mb of cAus introgression that we considered to study the origin of the cAus introgression. The darkred dashes lines indicate the 100kb of right border of cAus introgression identified between 25.8 and 26.7 Mb.

Mixture generation (mg) = 20

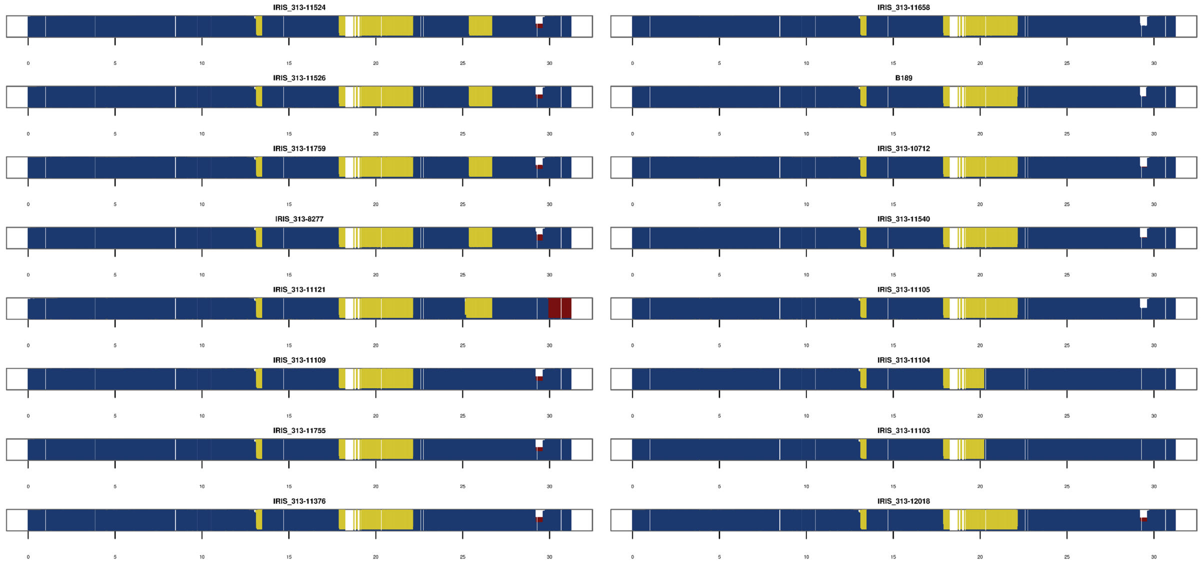

Figure S4. ELAI result in chromosome 6 with parameter mg=20. The number of generations since hybridization is represented by the parameter mg. This parameter influences the smoothness of the local ancestry inference and is taken into consideration during switching between the different layers of the HMM model.

Mixture generation (mg) = 50

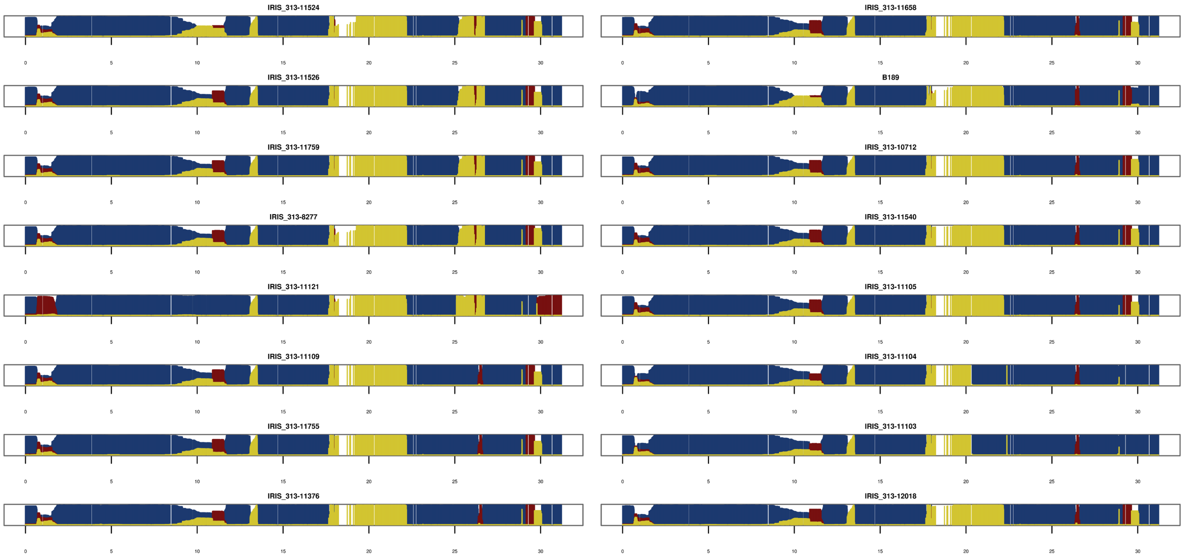

Figure S5. ELAI result in chromosome 6 with parameter mg=50

Mixture generation (mg) = 100

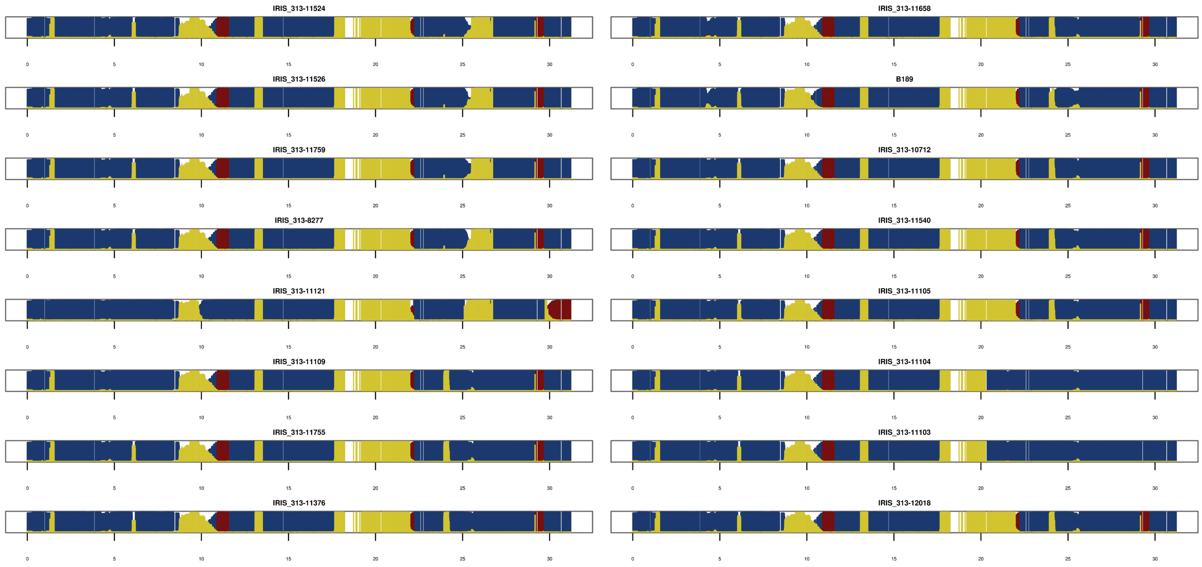

Figure S6. ELAI result in chromosome 6 with parameter mg=100

Mixture generation (mg) = 1000

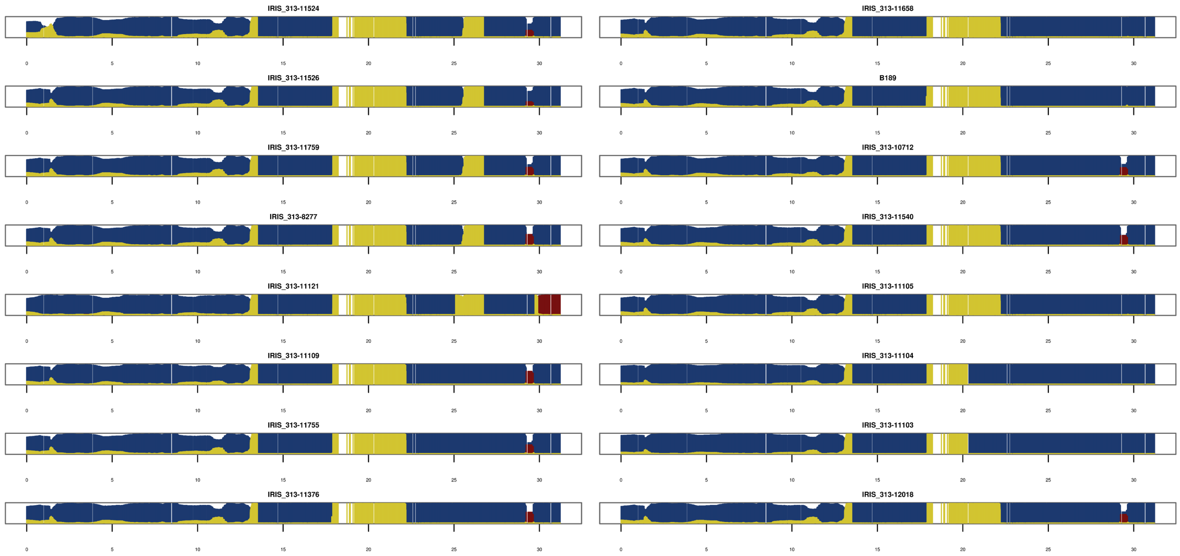

Figure S7. ELAI result in chromosome 6 with parameter mg=1000

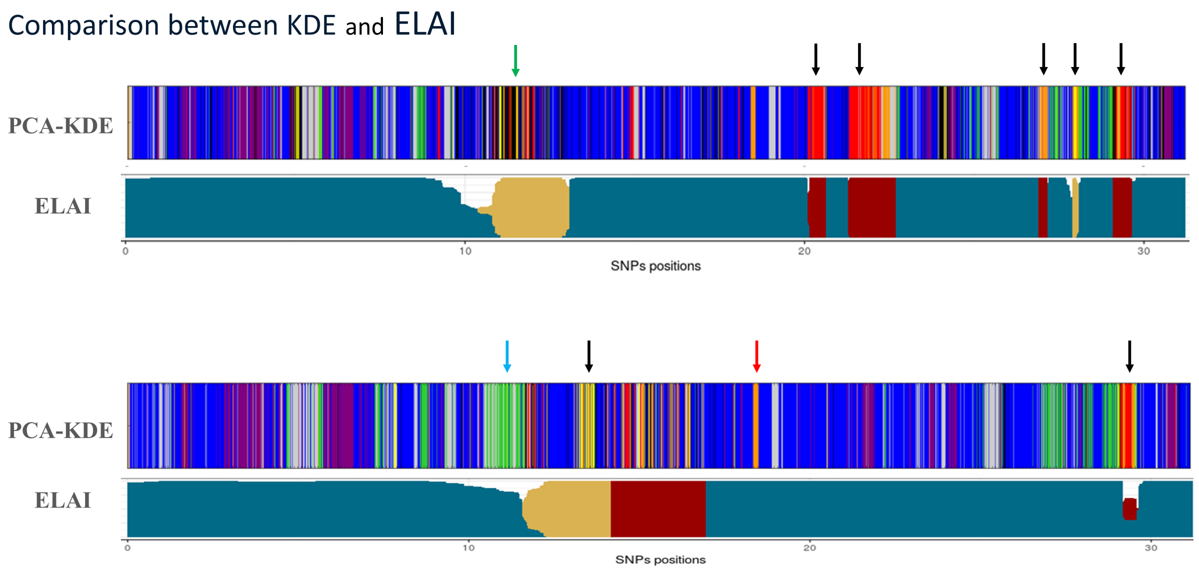

Figure S8. Comparison betwenn PCA-KDE and ELAI results. This graphic depicts some surveys that compare the findings of the two methodologies. These are two distinct chromosomes from two individuals chosen at random. The black arrows show where the two approaches obtain the same structures. A section painted in orange is indicated by the red arrow at the top. The orange is a haplotype present in both Indica and cAus plants that is the sum of yellow and red. The blue arrow highlights a region (green) indicated by the PCA-KDE method to be admixed between japonica (blue) and cAus (yellow), but ELAI reveals a greater allelic dose of Japonica than cAus. Both approaches find more or less the same thing, but with a different reading depending on the method. Finally, the green arrow indicates an area whose alleles are estimated to be cAus by ELAI but which PCA-KDE has difficulties appropriately assigning. The dark colour on the PCA-KDE colour map represents haplotypes with no alleles detected among the individuals representing the ancestral poles.

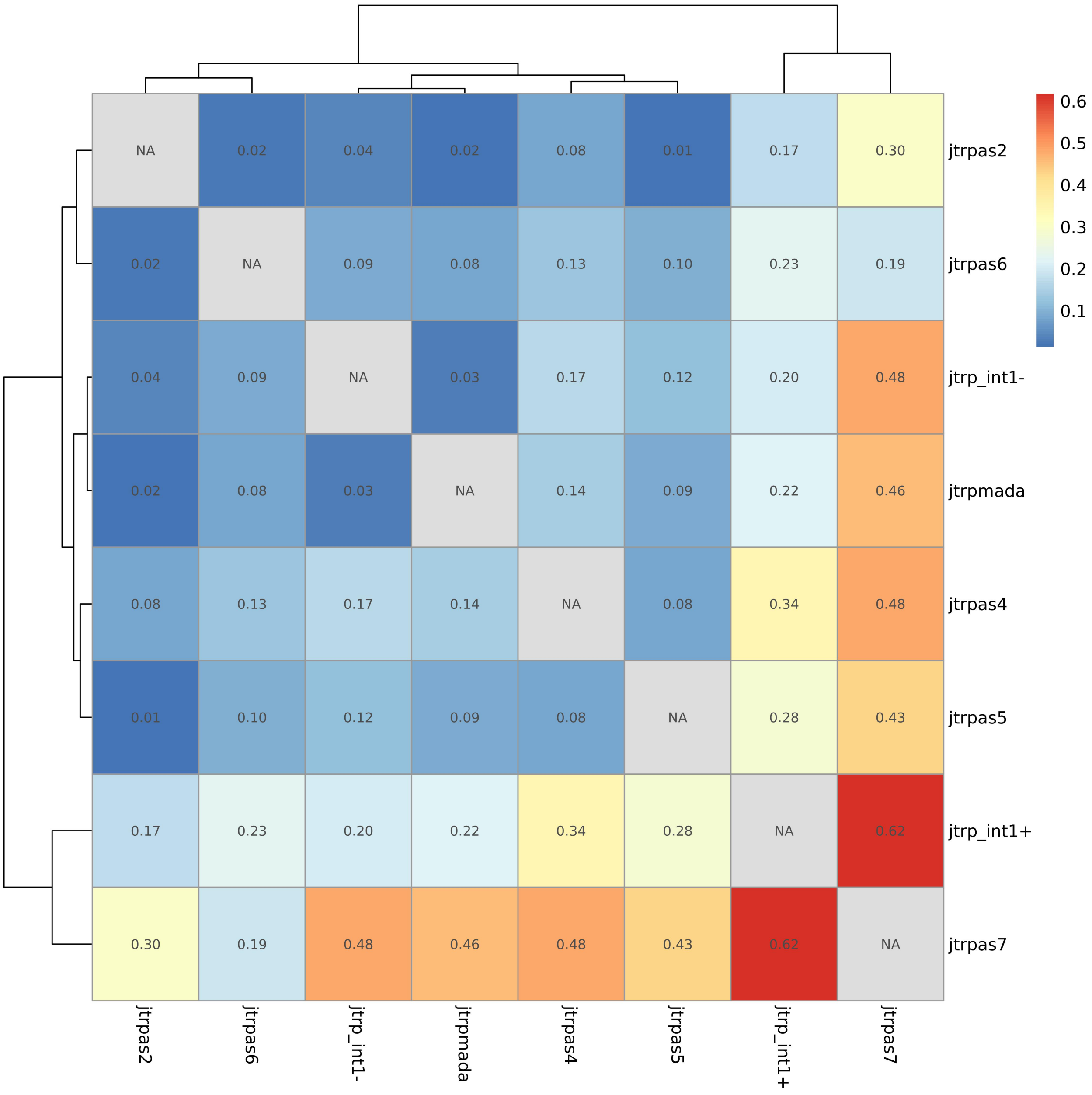

Figure S9. The FST pairwise analysis of tropical Japonica accessions from Asia and Africa from distinct geographical groupings. The whole genome was analysed in this study. The values inside the boxes indicate the Fst value of the two populations that intersect within the box. The shade of the boxes reflects the amount of differentiation associated with the FST value.

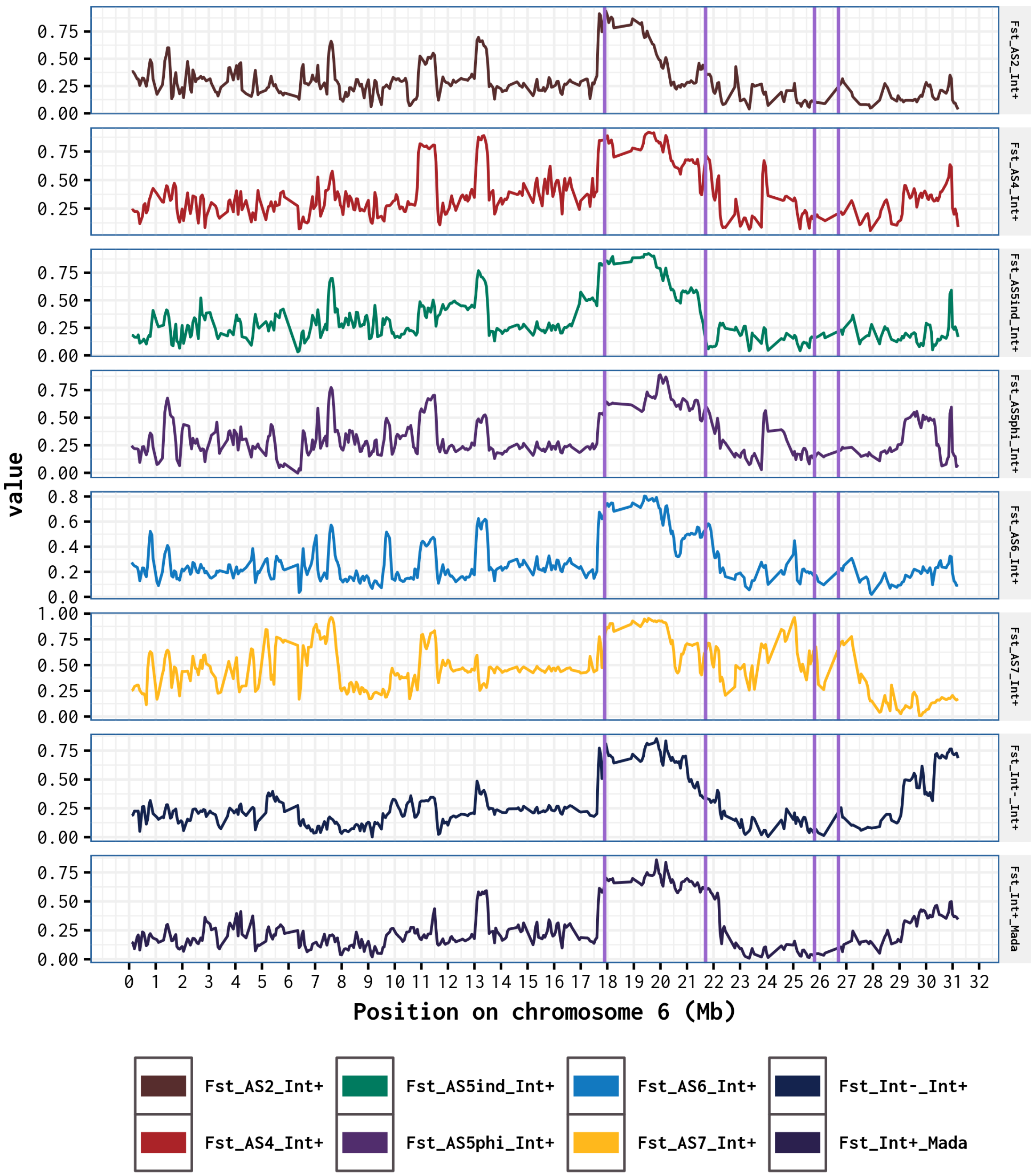

Figure S10. Local sliding FST analysis along chromosome 6 comparing Int+ *vs* other Japonica tropical subgroup from Asia and Africa. Calculations are performed on 100 kb windows overlapping by 50 kb. Each graph corresponds to a comparison between populations. The vertical purple bands delineate the locations of the Int1 (17.9-21.7 Mb) and Int2 (25.8-26.7 Mb) introgressions of cAus on chromosome 6 of Upland West African accessions respectively.

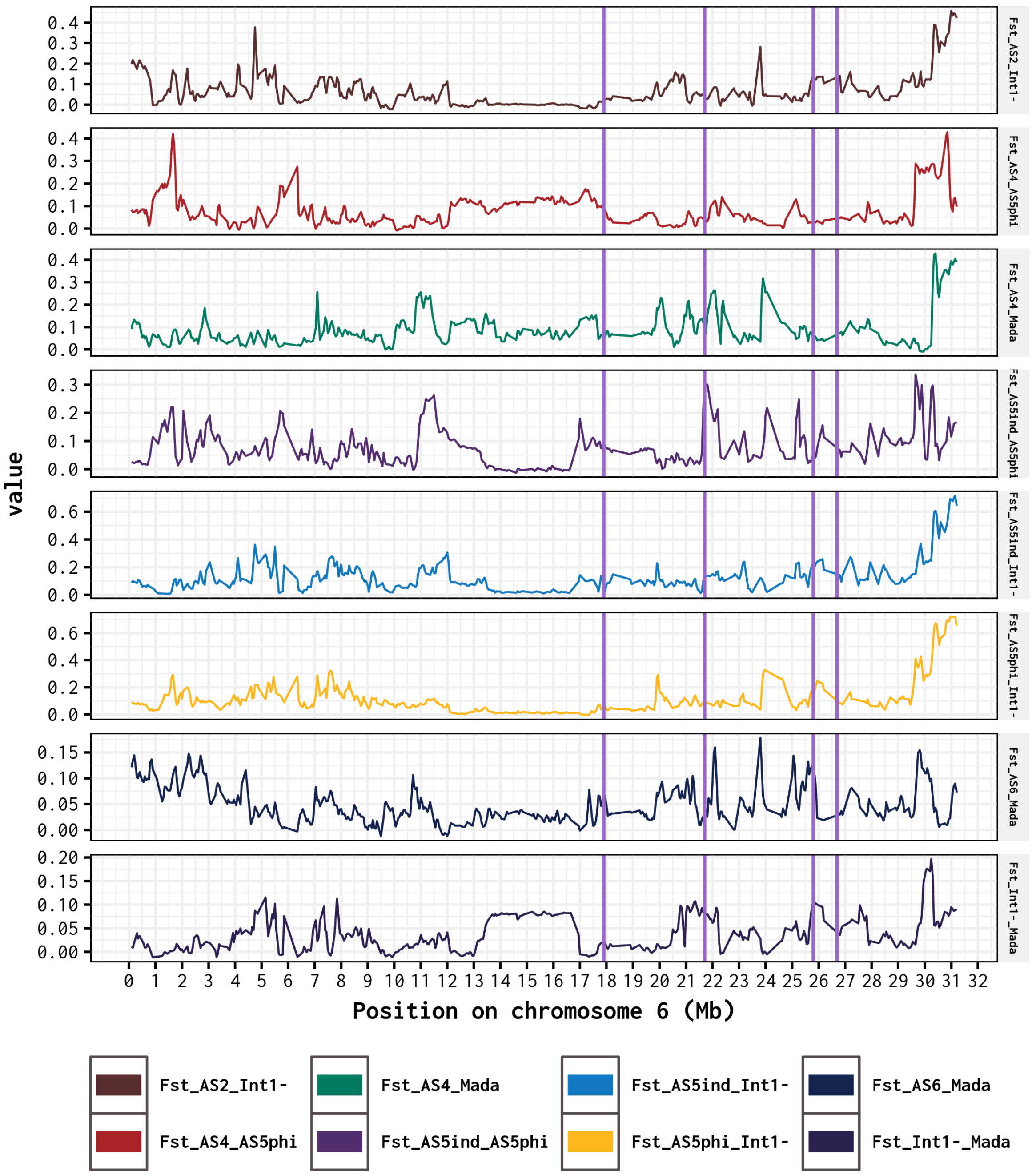

Figure S11. Local sliding FST analysis along chromosome 6 between different tropical Japonica populations from Asia and Africa. Calculations are performed on 100 kb windows overlapping by 50 kb. Each graph corresponds to a comparison between populations. The vertical purple bands delineate the locations of the Int1 (17.9-21.7 Mb) and Int2 (25.8-26.7 Mb) introgressions of cAus on chromosome 6 of Upland West African accessions respectively.

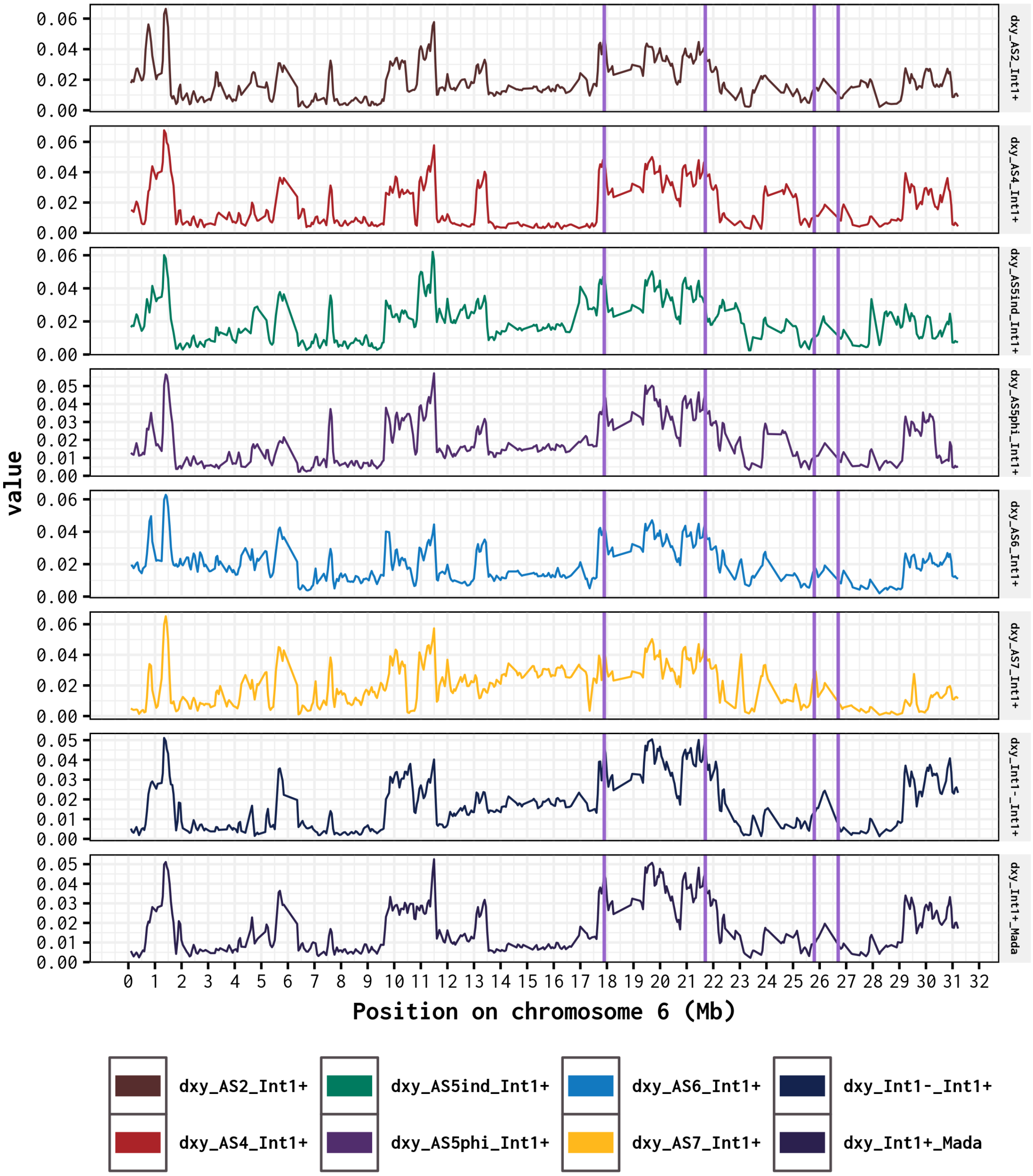

Figure S12. Local sliding genetic sequence divergence analysis (dxy) along chromosome 6 between different tropical Japonica populations between Asia and Africa. Calculations are performed on 100 kb windows overlapping by 50 kb. Each graph corresponds to a comparison of tropical upland rice accessions with cAus introgression from West Africa *vs* other tropical Japonica populations. The vertical purple bands delineate the locations of the Int1 (17.9-21.7 Mb) and Int2 (25.8-26.7 Mb) introgressions of cAus on chromosome 6 of Upland West African accessions respectively.

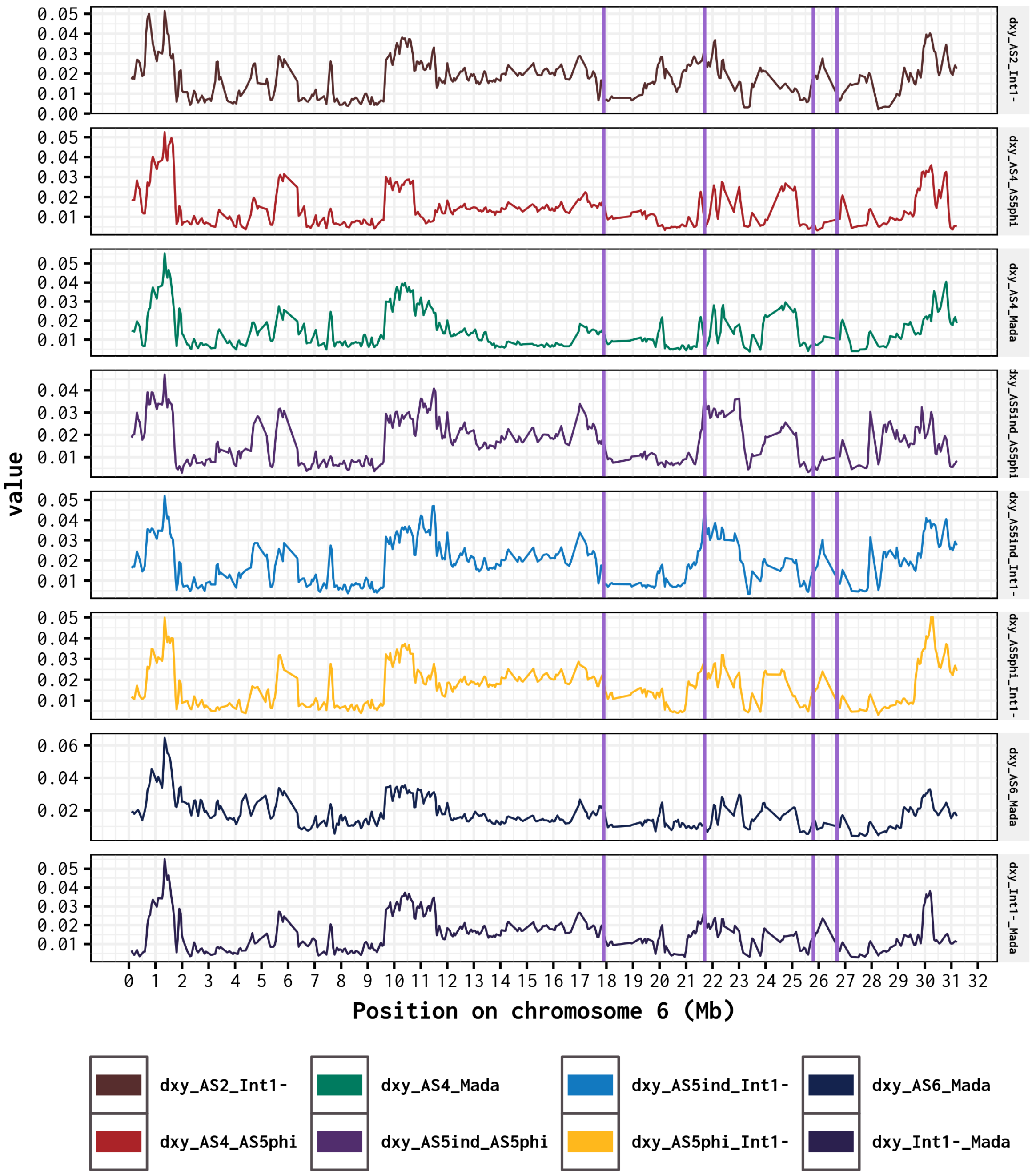

Figure S13. Local sliding genetic sequence divergence analysis (dxy) along chromosome 6 between different tropical Japonica populations from Asia and Africa. Calculations are performed on 100 kb windows overlapping by 50 kb. Each graph corresponds to a comparison between populations. The vertical purple bands delineate the locations of the Int1 (17.9-21.7 Mb) and Int2 (25.8-26.7 Mb) introgressions of cAus on chromosome 6 of Upland West African accessions respectively.

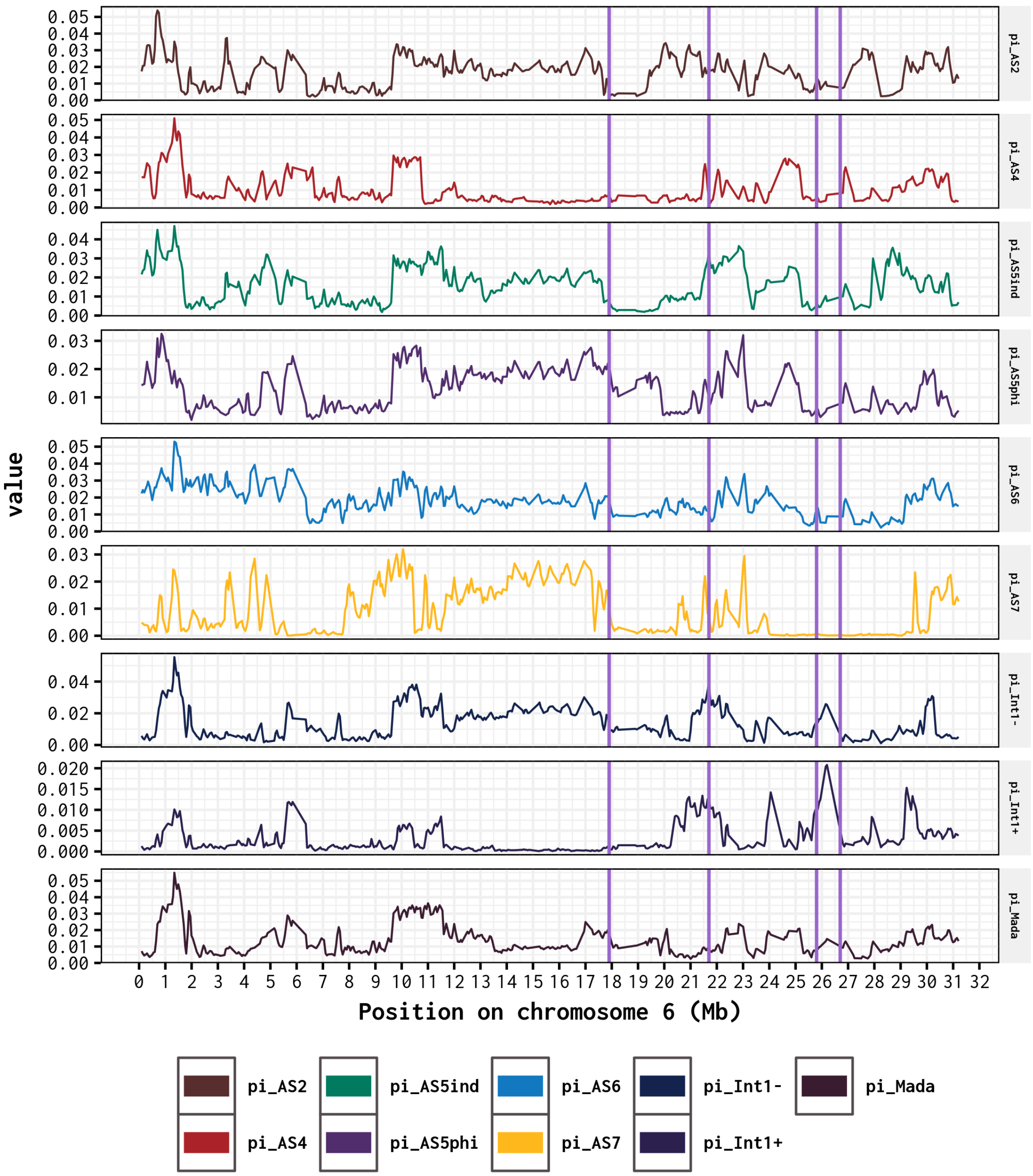

Figure S14. Local sliding genetic variation analysis (Pi) along chromosome 6 between different tropical Japonica populations between Asia and Africa. Calculations are performed on 100 kb windows overlapping by 50 kb. The vertical purple bands delineate the locations of the Int1 (17.9-21.7 Mb) and Int2 (25.8-26.7 Mb) introgressions of cAus on chromosome 6 of Upland West African accessions respectively.

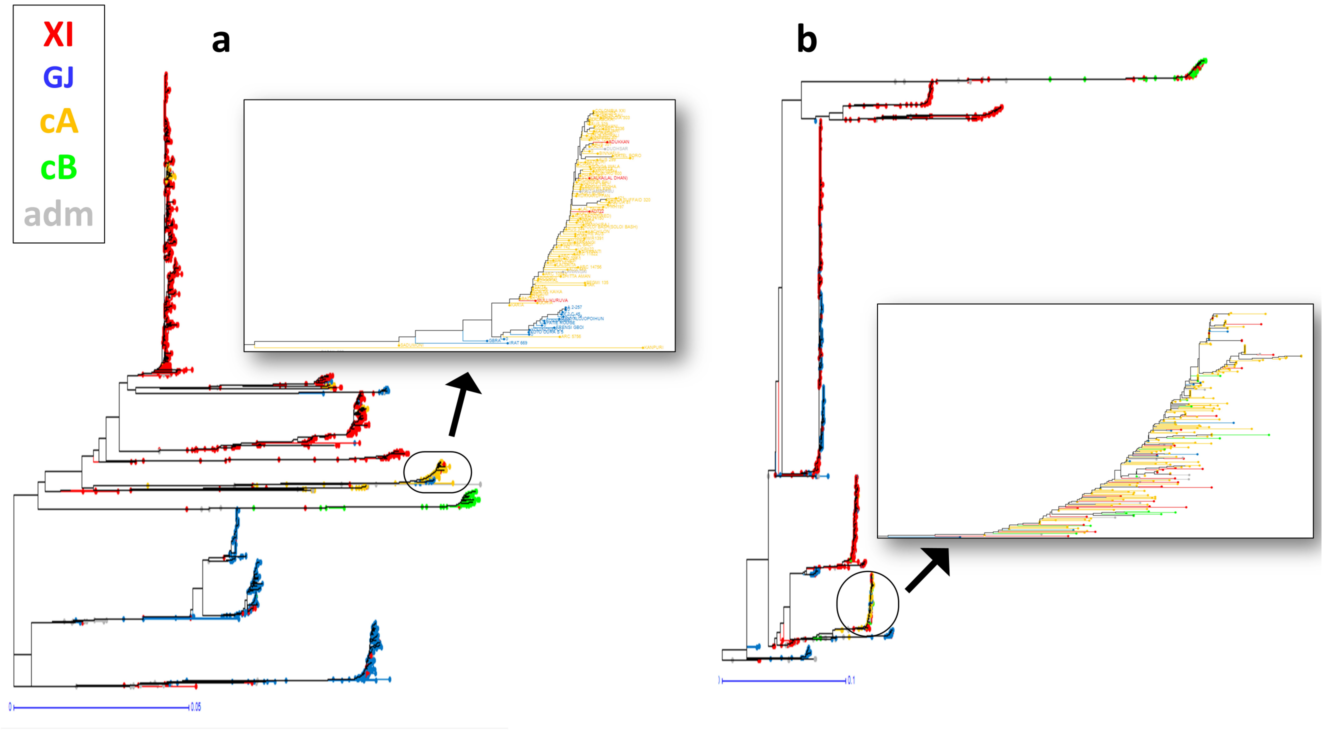

Figure S15. (a) Neighbor joining tree based on dissimilarities among the 3K accessions calculated from 1577 contiguous SNP loci located between 17.9 and 18.0 Mb on chromosome 6. This represents the inner left border of the large introgression (Int1) of cA origin in the African upland cultivars. (b) Neighbor joining tree based on dissimilarity among the 3K accessions calculated from 1344 contiguous SNP loci located between 21.6 and 21.7 Mb on chromosome 6. This represents the inner right border of the large introgression (Int1) of cA origin in the African upland cultivars.

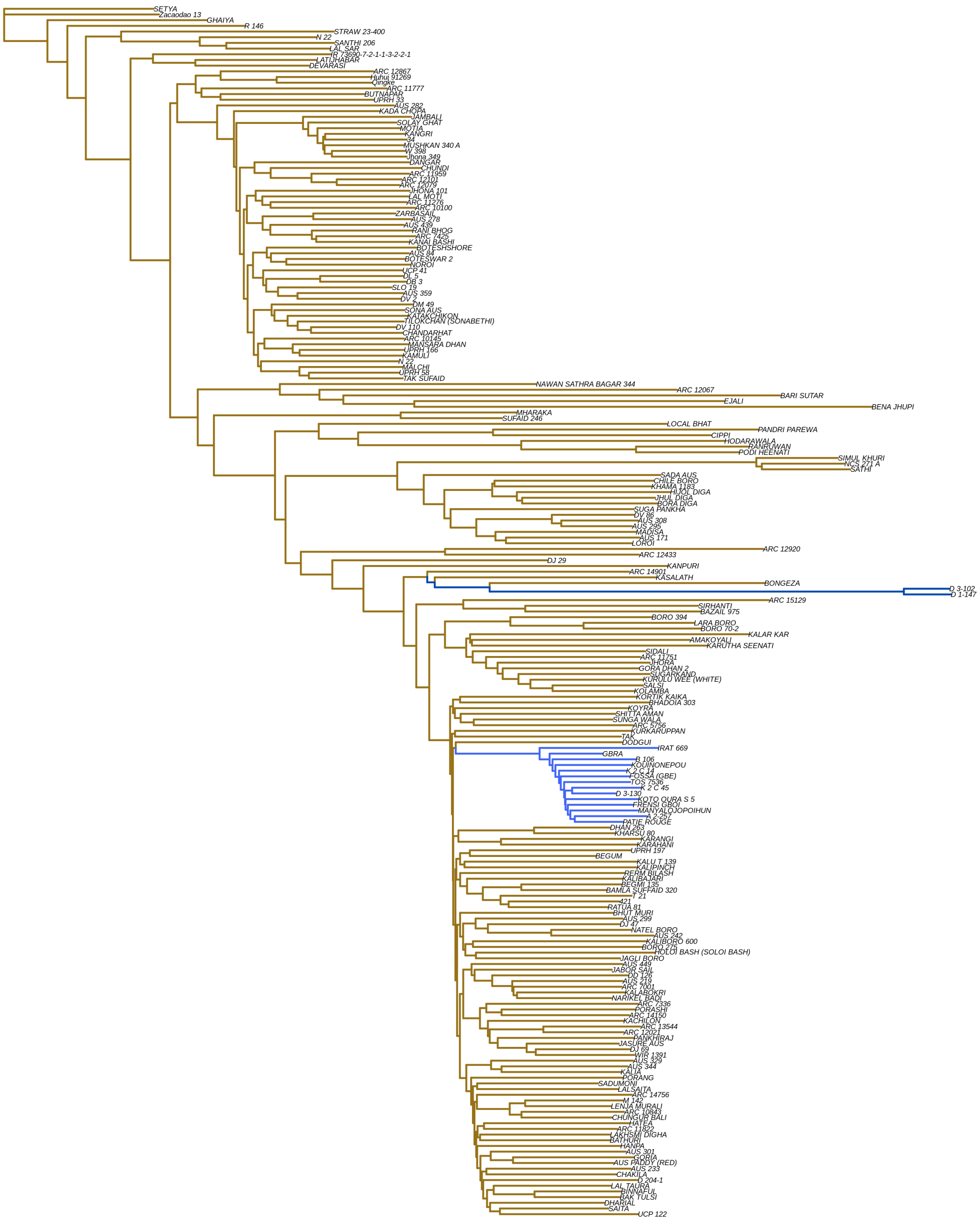

Figure S16. The UNJ tree was built using 3.8 Mb (17.9 - 21.7 Mb) in chromosome 6 between tropical West African japonica with cAus introgression and all Asian cAus.

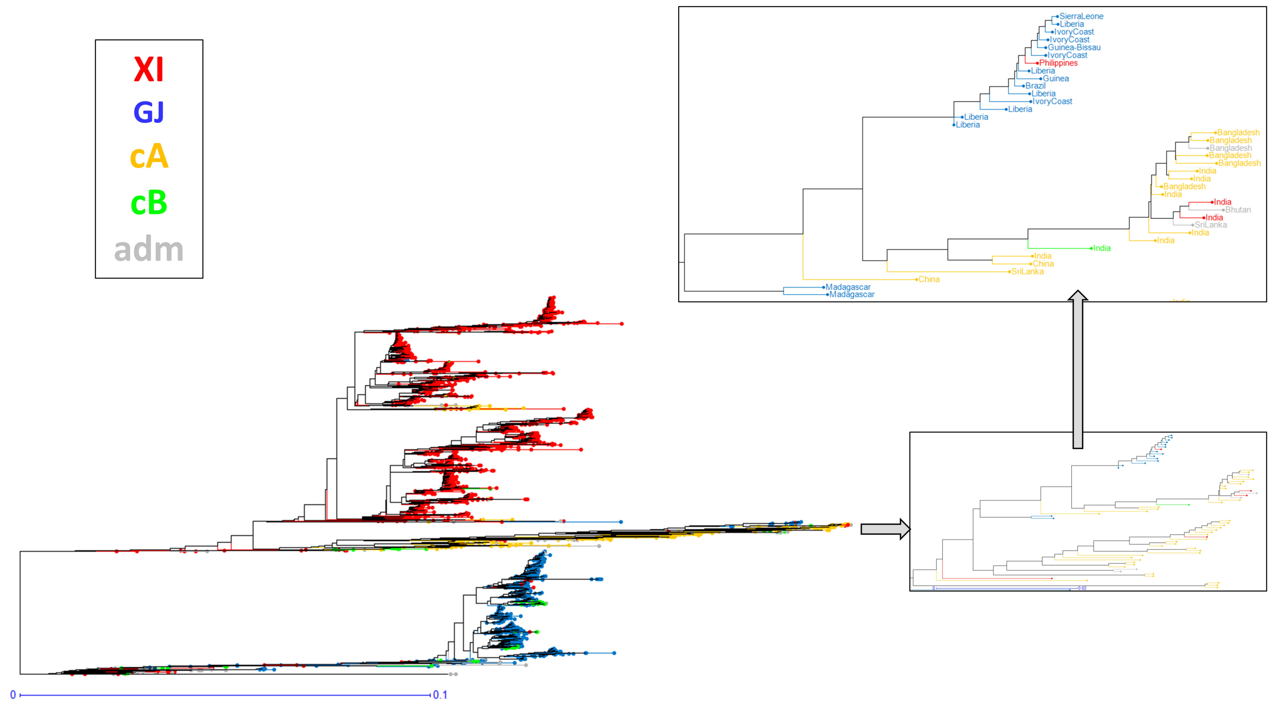

Figure S17. Neighbour-joining tree constructed using 900Kb located between 25.8 and 26.7 Mb on chromosome 6. The colour coding corresponds to Japonica (blue), Indica (red), cAus (yellow), Basmati (green) and admix (grey).

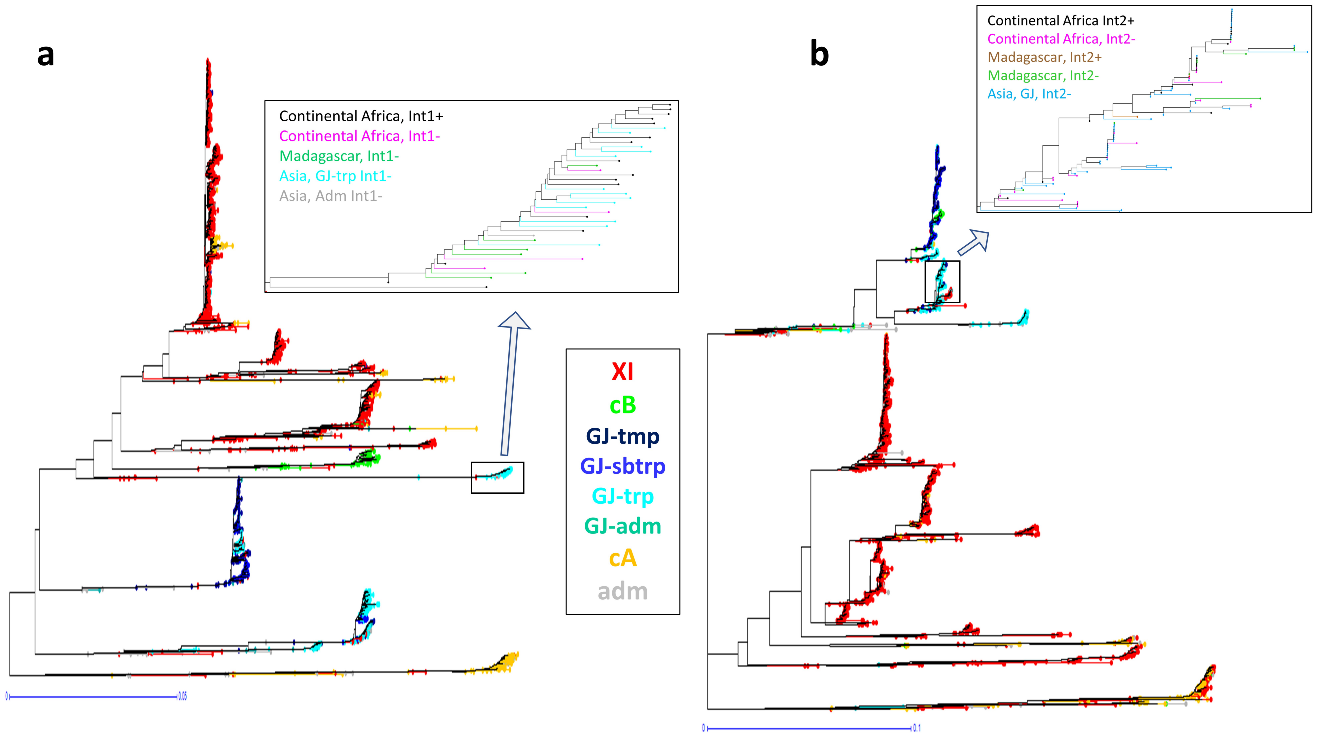

Figure S18. (a) Neighbor joining tree based on dissimilarities among the 3K accessions calculated from 2148 contiguous SNP loci located between 17.7 and 17.8 Mb on chromosome 6. This represents the outer left border of the large introgression (Int1) of cA origin in the African upland cultivars. Only 2998 accessions with less than 10% missing data were used for the tree. (b) Neighbor joining tree based on dissimilarity among the 3K accessions calculated from 929 contiguous SNP loci located between 26.75 and 26.85 Mb on chromosome 6. This represents the outer right border of the small introgression (Int2) of cA origin in the African upland cultivars.

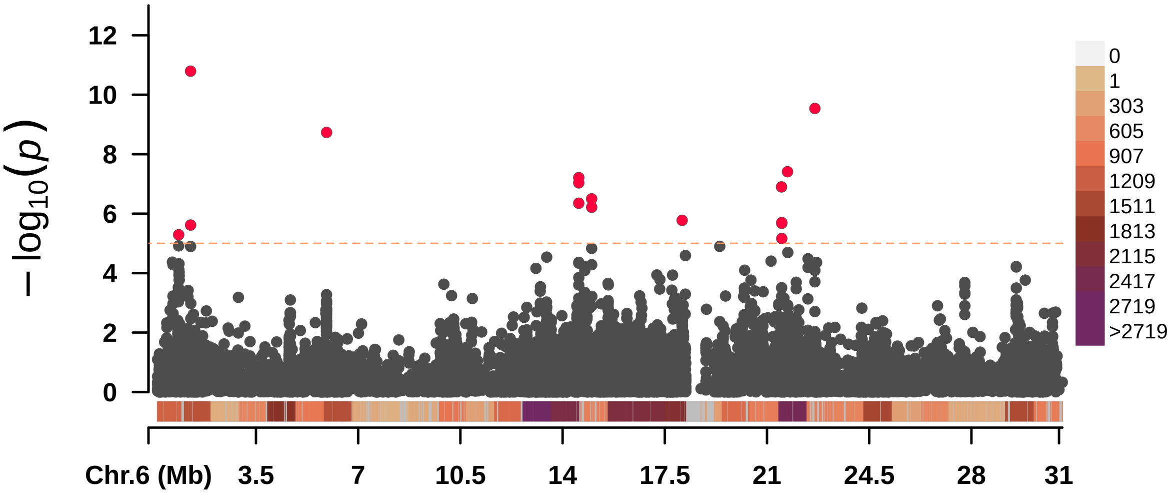

Figure S19. Manhattan plot on chromosome 6 of Asian upland accessions. The following graph shows the iHS genomic scan results for the identification of evidence of selection. The line orange corresponds to the significant threshold. The right legend shows the SNPs density for each 1 MB window.

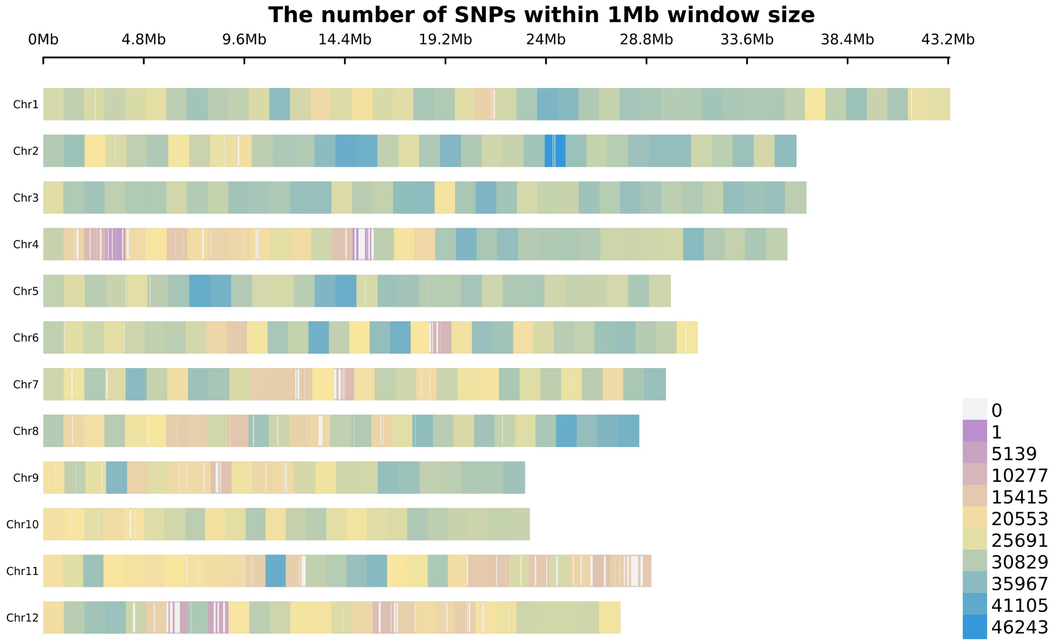

Figure S20. Distribution of the 10 million SNP markers across the 12 chromosomes of the rice genome. SNP density was calculated for each 1Mb window. This graph is drawn in Rstudio using the CMplot package available at https://github.com/YinLiLin/CMplot.
